# Impact of inter-partner HIV disclosure patterns in Malawi’s PMTCT program: A mixed-method study

**DOI:** 10.1371/journal.pone.0219967

**Published:** 2019-07-26

**Authors:** Monique van Lettow, Fabian Cataldo, Megan Landes, Florence Kasende, Pearson Nkhoma, Joep J. van Oosterhout, Evelyn Kim, Erik Schouten, Ernest Nkhoma, Rose Nyirenda, Beth A. Tippett Barr

**Affiliations:** 1 Medical and Research Department, Dignitas International, Zomba, Malawi; 2 Dalla Lana School of Public Health, University of Toronto, Toronto, Canada; 3 Department of Family and Community Medicine, University of Toronto, Toronto, Canada; 4 Department of Medicine, College of Medicine University of Malawi, Blantyre, Malawi; 5 United States Centers for Disease Control and Prevention, Lilongwe, Malawi; 6 Management Sciences for Health, Lilongwe, Malawi; 7 Malawi Ministry of Health, Lilongwe, Malawi; 8 United States Centers for Disease Control and Prevention, Kisumu, Kenya; The Ohio State University, UNITED STATES

## Abstract

**Background:**

Evidence suggests that disclosure of HIV status between partners may influence prevention of maternal-to-child transmission of HIV (PMTCT) outcomes. We report partner disclosure in relation to maternal antiretroviral therapy (ART) uptake and adherence, and MTCT among postpartum HIV-infected Malawian women.

**Methods:**

A cross-sectional mixed-method study was conducted as part of a nationally representative longitudinal cohort study. Between 2014–2016, all (34,637) mothers attending 54 under-5 clinics with their 4–26 week-old infants were approached, of which 98% (33,980) were screened for HIV; infants received HIV-1 DNA testing. HIV-exposure was confirmed in 3,566/33,980 (10.5%). Baseline data from mothers who were known to be HIV-infected at time of screening were included in the current analysis. Guardians (n = 17), newly diagnosed HIV-infected mothers (n = 256) and mothers or infants with undetermined HIV status (n = 30) were excluded. Data collected included socio-demographics, partner disclosure, maternal ART uptake, and adherence. Between 2016–2017, in-depth interviews and focus group discussions were conducted with adult mothers (n = 53) and their spouse/cohabiting partners (n = 19), adolescent mothers (n = 13), lost-to-follow up (LTFU) mothers (n = 22), community leaders (n = 23) and healthcare workers (n = 154).

**Results:**

Of 3153 known HIV-infected mothers, 2882 (91.4%) reported having a spouse/cohabiting partner. Among 2882 couples, both partners, one partner, and neither partner disclosed to each other in 2090 (72.5%), 622 (21.6%), and 169 (5.9%), respectively. In multivariable models, neither partner disclosing was associated with no maternal ART (aOR 4.7; 95%CI 2.5–8.8), suboptimal treatment adherence (aOR 1.8; 95%CI 1.1–2.8) and MTCT (aOR 2.1; 95%CI 1.1–4.1). Women’s fear of blame by partners was central to decisions not to disclose within couples and when starting new relationships. LTFU mothers struggled to accept and disclose their status, hindering treatment initiation; some were unable to hide ART and feared involuntary disclosure.

**Conclusion:**

Partner disclosure seems to play an important role in women’s decisions regarding ART initiation and adherence. Inter-partner non-disclosure was associated with no ART uptake, suboptimal treatment adherence and MTCT.

## Introduction

Global efforts to prevent mother-to-child transmission of HIV (MTCT) currently focus on expanding antiretroviral therapy (ART) coverage among HIV-infected pregnant mothers and ensuring maternal retention in care and viral suppression throughout the pregnancy, delivery and breastfeeding periods [1]. In 2011, Malawi was the first country to develop and implement ‘Option B+’, a universal test and treat strategy of lifelong ART for all pregnant and breastfeeding women. Since implementation, the Malawi Ministry of Health has documented marked increases in uptake of ART among women, yet programmatic effectiveness depends on ensuring adequate adherence to treatment and retention of women in care, which remains suboptimal [2].

Male partner involvement has been shown to play a role in improving women’s engagement in prevention of MTCT (PMTCT) [3–7]. Recent studies demonstrate that male partner involvement can increase women’s likelihood of accepting HIV testing, initiating ART, and remaining in care, ultimately reducing the risk of infant transmission and death [8–10]. However, an initial step towards male partner engagement is the woman’s disclosure of her own HIV status to her partner. In addition, mutual disclosure where both partners disclose their positive or negative status to each other is likely to promote treatment initiation and strengthen retention in care. Women’s fear of disclosure to their partner has been shown to negatively impact maternal uptake and adherence to ART [11–13] and further studies demonstrate that women who have disclosed achieve better PMTCT outcomes in the context of Option B+ [14–16].

While benefits of maternal disclosure are well documented, outcomes from our nationally-representative cohort in Malawi’s Option B+ program show an increased risk of early transmission of HIV (4–12 weeks) among women whose partner had not disclosed his own HIV status to them, which suggests complex dynamics between partner disclosure, partner’s HIV and ART status, and their respective impacts on PMTCT outcomes [17]. Overall, limited data exist in sub-Saharan Africa regarding disclosure patterns within couples and the impact on PMTCT utilization in the era of Option B+. Studies in the region tend to report on maternal disclosure alone; estimates of maternal disclosure in Option B+ programmes ranged from 69–77% [14, 15]. We have found no estimates of between-partner disclosure or knowledge of one’s partners’ HIV status and their impact on PMTCT outcomes.

In this paper, we utilized a mixed-methods approach to describe partner disclosure patterns among women at 4–26 weeks postpartum in the Malawi Option B+ PMTCT program, and to explore how knowledge of a partner’s HIV status and between-partner disclosure might impact uptake and adherence to maternal ART and infant transmission.

## Methods

### Study setting

Implementation of the Malawi integrated PMTCT/ART guidelines began in July 2011, giving all pregnant and breastfeeding women identified as HIV-infected in antenatal and maternal clinics access to life-long ART. At time of HIV status ascertainment, HIV-infected women receive 6 weeks of nevirapine prophylaxis with instruction to give this to their infants from birth, and were advised to bring their infants at six-weeks of age for virological testing at an under-5 clinic [18].

### Study design and participants

This is a cross-sectional mixed-methods study using baseline data of known HIV-infected mothers at 4–26 weeks postpartum who were enrolled for longitudinal follow up within the National Evaluation of the Malawi PMTCT Program (NEMAPP); NEMAPP methods are described in detail elsewhere [19]. Briefly, between October 2014 and May 2016, all mothers (or guardians, if the mother had died) with infants 4–26 weeks old attending an under-5 clinic in 54 randomly selected health facilities across 10 districts and four regional sampling zones (North-Central rural, North-Central urban, South rural and South urban) were screened for inclusion in the NEMAPP study. This age group was selected as it was determined from population based surveys and Malawi Ministry of Health data that it would allow the study to capture almost all infants attending for the first immunization visit; those missed at a first visit would still be identified at a subsequent visit.

Eligibility criteria for the mother-infant pair included confirmed HIV exposure in infants, infant age between 4 and 26 weeks at time of screening, and mother present at screening, or confirmed dead by legal guardian. Following national guidelines, a positive HIV rapid test in the mother or infant (if the mother had died) indicated infant HIV exposure [18]. Out of 34,637 mothers (or guardians) and their infant approached, 33,980 (98.1%) were screened for HIV, including 236 (0.7%) guardians of infants whose mothers were confirmed dead. A total of 30,281 were confirmed HIV-negative, 133 had inconclusive test results and 10 were excluded as a result of missing infant HIV test results (n = 2) or concerns about clerical errors in infant HIV test results (n = 8). HIV-exposure was confirmed in 3,566/33,980 (10.5%). A total of 3456 mothers (or guardians) with HIV-exposed infants were enrolled for the NEMAPP longitudinal follow up. Baseline data from mothers who were known to be HIV-infected at time of screening were included in the current analysis. Guardians (n = 17), newly diagnosed HIV-infected mothers (n = 256) and mothers or infants with undetermined HIV status (n = 30) were therefore excluded.

Between July 2016 and September 2017, a representative sample of 13 out of the 54 health facilities was selected to conduct a longitudinal qualitative sub-study implemented over 15 months. Sites were identified across 4 geographical strata and were included in the NEMAPP 48-month extended cohort. Data were collected through two waves of data collection at 8-month intervals for all the study sites with the same individuals; In-depth interviews were conducted with a subgroup of adult mothers (n = 53) and their partners (n = 19), adolescent (10–19 year old) mothers (n = 13), and mothers who were lost-to-follow up (LTFU, i.e., more than two months overdue after a scheduled appointment and all efforts to locate the woman had been exhausted; n = 22). The larger study (from which data are not presented in this paper) also included the views of community leaders (n = 23) andfocus group discussions (FGDs) were conducted in each of the 13 health facilities with healthcare workers (HCWs) (n = 154).

### Data collection and laboratory procedures

For the quantitative study, mothers were interviewed by trained health facility staff at a private location in the clinic. Mothers were interviewed at enrolment on age, parity, time from when mother knew her HIV-positive status, partner’s HIV-status and disclosure status between partners, uptake of and adherence to maternal ART and uptake of infant nevirapine prophylaxis for HIV-exposed infants, using structured questionnaires. Quantitative data was based on self-reports and verified through health booklets and clinical records were possible. The sample of respondents engaged in the qualitative sub-study were interviewed on women’s barriers and facilitators to start and continue ART, disclosure, and family dynamics. Interviews and FGDs were conducted in private locations and audio recorded.

Dried blood spot (DBS) specimens from infants were tested in a reference laboratory. A qualitative HIV-1 DNA polymerase chain reaction test (COBAS AmpliPrep/COBAS TaqMan Qualitative Assay, version 2.0, Roche Diagnostics, Indianapolis, IN, USA) was performed on all HIV-exposed infant DBS samples to determine whether the infant was HIV-infected.

### Study definitions

All mothers were asked whether they currently had a spouse or cohabiting male partner, and among those with a partner, whether they had disclosed their HIV status to their partner and if they knew his HIV status.

‘Both partners disclosed (any status)’ was defined as the mother disclosed her HIV-positive status to her partner and knew his HIV status. ‘One partner disclosed (any status)’ was defined as the mother disclosed her HIV-positive status to the partner but did not know his HIV status or the mother did not disclose her HIV-positive status to the partner, but knew his HIV status. ‘Neither partner disclosed’ was defined as the mother did not disclose her HIV-positive status to her partner and did not know his HIV status.

Current uptake of and adherence to maternal ART were recorded as reported by the mother. Whenever possible, interviewers checked the mothers’ health booklets to check the accuracy of the mothers’ responses. Among those on ART, and in alignment with the Malawi Ministry of Health guidelines, optimal treatment adherence was defined as self-reported missing none or one day of once daily fixed-dose combination antiretrovirals (ARVs), and suboptimal treatment adherence as self-reported missing ≥2 days of ARVs in the last month. Uptake (any number of days given from birth) and missing nevirapine prophylaxis syrup for exposed infants was recorded as reported by the mother.

We calculated MTCT ratios at 4–26 weeks postpartum as the percentage of infants tested for HIV-1 DNA who were positive.

### Analysis

Descriptive statistics were used to characterize study participants and estimate the proportion of each outcome. Characteristics were described with numbers and proportions or medians with interquartile ranges (IQR). To get further insight in the complex dynamics between partner disclosure, partner’s HIV and ART status, and their respective impacts on PMTCT outcomes, we explored the following associations: i) having a partner or not, ii) being in a relationship in which the mother disclosed or not, iii) being in a relationship in which the partner disclosed or not, iv) being in a relationship with a partner whose HIV status is positive, negative, or unknown/non-disclosed, and v) being in a relationship in which both, one, or neither partner(s) disclosed their HIV status, separately with missing uptake of maternal ART, suboptimal treatment adherence, and MTCT. Multivariable logistic regression analysis was used to calculate adjusted odds ratios (aOR) with 95% confidence intervals (CI) for each model and with adjustment for geographic region, age, parity, and time from when mother knew her HIV-positive status (prior to, during, or after the index pregnancy). We also adjusted for uptake of maternal ART and infant nevirapine prophylaxis in the models for MTCT. All variables were simultaneously entered in the logistic regression model as the first step and tested for removal one by one (p-value cut-off 0.05). Analyses were conducted using IBM SPSS Statistics 24 (IBM, Armonk, NY, USA). Overall MTCT, and MTCT ratios among mothers who were in a relationship where both, one, or neither of the partners disclosed were reported with 95% CI.

Qualitative data were fully transcribed and translated from Chichewa to English. All data were coded using *NVivo11* (QSR International, Melbourne, Australia). Four coders, including one investigator and three field researchers, coded the qualitative data and reached consensus on a common analysis framework and associated coded extracts. Inductive and deductive analyses were used to identify common themes across the different categories of informants, which were organized through a content analysis approach to compare responses between two waves of data collection at 8-month interval. The framework analysis followed emerging categories from the dataset in relation to decision making and patient agency; barriers and facilitators to uptake and retention; perception of ART; experience of breastfeeding and pregnancy; health staff perception; and disclosure of HIV status.

### Ethics approval and consent to participate

Ethical approval for the study was provided by Malawi’s National Health Sciences Research Committee (#1262 and #1381), the Centers for Disease Control and Prevention (CDC) Center for Global Health Associate Director of Science (#2014-054-7 and #2016–133), and the University of Toronto Research Ethics Board (#30448). All participants provided written or witnessed thumbprint informed consent.

## Results

A total of 3153 mothers reported knowing that they were HIV-infected at the time of study screening and were included in the analyses; 1593 (50.5%) knew their positive status from before the index pregnancy, 1512 (48.0%) from being tested positive during the index pregnancy, and 48 (1.5%) from being tested positive after the index pregnancy.

Table 1 describes the characteristics of the mothers included in this study. Mothers’ median age was 30 years (IQR 25–34) and median parity was three children (IQR 2–5).

**Table 1:** Characteristics of postpartum HIV-infected mothers.

	*n*	% or median (IQR)
**Total**	***3153***	
**Geographical Region**		
North/central rural	*785*	24.9
North/central urban	*828*	26.3
South rural	*868*	27.5
South urban	*672*	21.3
**Mother’s age in years, median (IQR)**	*3142*	30 (25–34)
**Mother’s age in years, %**		
≤19	*178*	5.6
20–24	*596*	18.9
25–29	*775*	24.6
≥ 30	*1593*	50.5
Missing	*11*	0.3
**Parity, median (IQR)**	*3146*	3 (2–5)
**Parity, %**		
1	*376*	11.9
2–3	*1304*	41.4
∥ 4	*1466*	46.4
Missing	*7*	0.2
**Known HIV-infected since:**		
Before index pregnancy	*1593*	50.5
During index pregnancy	*1512*	48.0
After index pregnancy	*48*	1.5
**Spouse/cohabiting partner**		
Has partner	*2882*	91.4
No partner	*265*	8.4
Missing	*6*	0.2
**Disclosure from mother to partner**^a^		
Mother disclosed	*2696*	93.5
Mother did not disclose	*186*	6.5
**Disclosure from partner to mother**^a^		
Partner disclosed HIV status (any) to mother^b^	*2107*	73.1
Partner did not disclose^c^	*774*	26.8
Missing	*1*	0.0
**Partner status reported by mother**^a^		
Partner is known HIV positive	*1491*	51.7
Partner is reported HIV negative	*616*	21.4
Partner’ HIV status unknown	*774*	26.9
Missing	*1*	0.0
**Disclosure between partners**^a^		
Both partners disclosed	*2090*	72.5
One partner disclosed	*622*	21.6
Neither of the partners disclosed	*169*	5.9
Missing	*1*	0.0
**Maternal ART status**		
On ART	*3053*	96.8
Started but stopped ART	*28*	0.9
Unknown/did not want to reveal	*44*	1.4
Did not start ART	*28*	0.9
**Number of days having missed ART in the last month among those on ART**		
0	*2460*	80.6
1 day	*250*	8.2
≥2 days	*317*	10.4
Missing	*26*	0.9
**Infant nevirapine prophylaxis received**		
Yes, received any days from birth	*3001*	95.2
Missed nevirapine prophylaxis uptake	*152*	4.8
**Exposed infants HIV status at 4–26 weeks (MTCT)**		
HIV-uninfected	*3058*	97.0
HIV-infected	*95*	3.0

HIV: human immunodeficiency virus

IQR: interquartile range

ART: antiretroviral therapy

MTCT: maternal-to-child transmission

^a^Among those with partner

^b^Mother reported knowing the result of partner’s HIV test if partner was ever tested

^c^Mother reported that partner never had an HIV test or did not know whether he had ever been tested

Among all mothers, 2882 (91.4%) reported having a spouse or cohabiting partner. Of these mothers, 2696 (93.5%) reported having disclosed their HIV status to their male partner and 2107 (73.1%) reported that their partner had disclosed to them (1491 partners were reported to be HIV positive and 616 HIV negative). Among the 2882 mothers with a partner, both partners disclosed in 2090 (72.5%) cases, one partner disclosed in 622 couples (21.6%; 605 mothers only and 17 partners only), and neither of the partners disclosed in 169 (5.9%) cases. Data were missing for one woman. Overall, 3053 (96.8%) mothers were on ART. Among the remaining 100 (3.2%) mothers, 44 unknown/did not want to reveal whether they were on ART, 28 had started but stopped, and 28 never started ART. Among the mothers on ART, 2460 (80.6%) reported no missed doses, 250 (8.2%) missed one day and 317 (10.4%) missed at least two days of ARVs in the last month. Infant nevirapine prophylaxis was received by 3001 (95.2%) of the 3153 exposed infants. At study enrolment, 95 (3.0%; 95% CI 2.4–3.6) exposed infants were HIV infected (MTCT). Young mothers (<19 and 20–24) were more likely to have no partner (<19 years of age: aOR 3.0; 95% CI 1.9–4.6), not have disclosed their status when having a partner (<19 of age: aOR 2.2; 95% CI 1.2–3.9, 20–24 years of age: aOR 1.5; 95% CI 1.01–2.3), and be in a relationship in which the partner had not disclosed his HIV status (<19: aOR 1.7; 95% CI 1.2–2.4, 20–24: aOR 1.3; 95% CI 1.1–1.7) than mothers over 30 years of age, when adjusted for geographical region, parity, and time from when the mother knew her positive status. Primiparous mothers were more likely to be in a relationship where neither of the partners disclosed their status (aOR 2.0; 95% CI 1.3–3.2) than mothers with at least four previous deliveries, when adjusted for geographical region, age, and time from when mother knew her positive status. (Data not shown)Table 2 shows the association between marital status, partner HIV status and between-partner disclosure status as they relate to the outcomes of maternal ART uptake, suboptimal maternal treatment adherence, and MTCT at 4–26 weeks postpartum.

**Table 2:** Partner disclosure patterns and associations with treatment and MTCT outcomes.

	Mother not on ART^a^				Suboptimal treatment adherence^b^				Mother to Child Transmission at 4–26 weeks			
	*n/N*^c^	%	aOR (95% CI)^f^	*p-value*	*n/N*^c^	*%*	aOR (95% CI)^f^	p-value	*n/N*^c^	%	aOR (95% CI) ^g^	p-value
**All**	*100/3153*	3.2			*318/3027*	10.5			*95/3153*	3		
**Marital status**												
Mothers with spouse/cohabiting partner	*84/2882*	2.9	**-**		*283/2779*	10.2	**-**		*81/2882*	2.8	**-**	
Mothers without a partner	*16/265*	6	2.4 (1.3–4.2)	0.003	*34/246*	13.8	1.4 (0.9–2.0)	0.11	*13/265*	4.9	1.7 (0.9–3.1)	0.09
Missing	*6-Jan*	16.7			*0/2*	0			*6-Jan*	16.7		
**Mother disclosure, among those with partner**												
Mother disclosed HIV-positive status to male partner	*62/2696*	2.3	-		*265/2615*	9.8	**-**		*67/2696*	2.5	**-**	
Mother did not disclose her status to male partner	*20/186*	10.8	3.6 (2.1–6.4)	0.0001	*27/164*	16.5	1.6 (1.0–2.4)	0.05	*14/186*	7.5	2.4 (1.3–4.4)	0.007
**Partner disclosure, among mothers with partner**												
Partner disclosed HIV status (any) to mother^d^	*38/2107*	1.8	**-**		*175/2053*	8.5	**-**		*55/2107*	2.6	**-**	
Partner did not disclose^e^	*45/774*	5.8	2.8 (1.5–5.2)	0.0001	*109/725*	15	1.8 (1.4–2.3)	0.001	*27/774*	3.5	1.1 (0.7–1.8)	0.76
Missing	*0/1*	0			*0/0*	0			*0/1*	0		
**Partner’s HIV status**												
Partner reported HIV positive	*24/1491*	1.6	**-**		*128/1454*	8.8	**-**		*34/1491*	2.3	**-**	
Partner reported HIV negative	*14/616*	2.3	1.5 (0.8–3.0)	0.25	*46/599*	7.7	0.9 (0.6–1.2)	0.45	*20/616*	3.2	1.4 (0.8–2.4)	0.25
HIV-status of partner unknown	*45/774*	5.8	3.2 (1.9–5.4)	0.001	*109/725*	15	1.7 (1.3–2.2)	0.001	*27/774*	3.5	1.2 (0.7–2.1)	0.44
Missing	*0/1*	0			*0/1*	0			*0/1*	0		
**Disclosure between partners**												
Both partners disclosed (any status)	*36/2090*	1.7	**-**		*171/2038*	8.4	-		*52/2090*	2.5	**-**	
One partner disclosed (any status)	*29/622*	4.7	2.5 (1.5–4.2)	0.001	*88/591*	14.9	1.8 (1.4–2.4)	0.001	*17/622*	2.7	0.9 (0.5–1.6)	0.76
Neither of the partners disclosed	*18/169*	10.7	4.7 (2.5–8.8)	0.0001	*24/149*	16.1	1.8 (1.1–2.8)	0.02	*12/169*	7.1	2.1 (1.1–4.1)	0.04
Missing	*0/1*	0			*0/1*	0			*0/1*	0		

ART: antiretroviral therapy

aOR: adjusted odds ratio

CI: confidence interval

^a^ Did not start, stopped or did not want to reveal

^b^ Missed at least two days of antiretroviral medication in the last month

^c^ Numerator/Denominator

^d^ Mother reported to know the result of HIV test ever taken by partner

^e^ Mother reported that partner never had an HIV test or did not know whether he ever had

^f^ Mother reported that partner never had an HIV test or did not know whether he ever had

^g^Adjusted for geographical region, age, parity, time from when mother knew her HIV-positive status, and uptake of maternal ART and infant nevirapine prophylaxis

In multivariable analysis that adjusted for geographical region, age, parity, and time from when the mother knew her positive status, mothers without a partner were more likely not to be on ART (aOR 2.4; 95% CI 1.3–4.2) than mothers with a partner.

Mothers who had not disclosed their HIV status to their partner were more likely not to be on ART (aOR 3.6; 95% CI 2.1–6.4) and have suboptimal treatment adherence (aOR 1.6; 95% CI 1.01–2.4) than mothers who had disclosed. They were also at higher risk of MTCT (aOR 2.4; 95% CI 1.3–4.4) when adjusted for geographical region, age, parity, time from when mother knew her positive status, uptake of maternal ART, and infant nevirapine prophylaxis.

Similarly, mothers with partners who had not disclosed to them were less likely to be on ART (aOR 2.8; 95% CI 1.5–5.2) and have suboptimal treatment adherence (aOR 1.8; 95% CI 1.4–2.3) than mothers with partners who had disclosed to them. Mothers with partners who were HIV status unknown (who had not disclosed) to them were more likely not to be on ART (aOR 3.2; 95% CI 1.9–5.4) and have suboptimal treatment adherence (aOR 1.7; 95% CI 1.3–2.2) than mothers with partners who were known to be HIV positive, when adjusted for geographical region, age, parity, and time from when mother knew her HIV-positive status.

Being in a relationship in which one or neither partner disclosed was associated with no maternal ART uptake (one partner only: aOR 2.5; 95% CI 1.5–4.2; neither of the partners: 4.7; 95% CI 2.5–8.8) and with suboptimal treatment adherence (one partner only: aOR 1.8; 95% CI 1.4–2.4; neither of the partners: aOR 1.8; 95% CI 1.1–2.8), when adjusted for region, age, parity, and time from when mother knew her HIV-positive status. Non-disclosure between partners was also associated with MTCT (aOR 2.1; 95% CI 1.1–4.1), when adjusted for region, age, parity, time from when mother knew her HIV-positive status, uptake of maternal ART, and infant nevirapine prophylaxis. MTCT among mothers who were in a relationship where both partners disclosed, one partner disclosed or neither of the partners disclosed was 2.5% (95% CI 1.8–3.2), 2.7% (95% CI 1.4–4.0) and 7.1% (95% CI 3.2–11.0), respectively. The MTCT rate among mother who were in a relationship where neither of the both partners disclosed was significantly higher than that among mothers who were in a relationship where both partners disclosed (p<0.01) or where one partner disclosed (p = 0.03).

Results from the qualitative sub-study illustrate some of the dynamics of disclosure between couples.

Table 3 summarizes patterns of disclosures for mothers involved in the sub-study and their reasons for lack of adherence to ART in relation to disclosure. The lack of disclosure between partners hindered, in some cases, the mother’s ability to continue treatment as some felt discouraged without their partner’s support.

**Table 3:** Disclosure amongst couples (qualitative only).

	Mothers without a partner	Both partners disclosed	Only mother disclosed to partner	Only male partner disclosed	Neither partner disclosed
*Adult Women*	0/53	40/53^a^	10/53	0/53	3/53
*Adolescents*	0/13	5/13	4/13^b^	0/13	4/13^c^
*Lost to Follow Up Women*	2/22	11/22^d^	6/22	0/22	3/22

^a^2/40 adult women in couples where both partners had disclosed experienced adherence challenges; one stopped antiretroviral treatment (ART) when she stopped breastfeeding, the other did not continue ART because she did not understand how she was infected with HIV.

^b^3/4 adolescents who disclosed to their partner did not adhere to ART (one was a sex worker finding it difficult to attend clinic visits, one was reluctant to be on ART because her partner refused to get tested, one did not want to continue ART after her partner refused to accompany her to the ART clinic).

^c^1/4 adolescents in couples where neither partner disclosed stopped ART because she feared inadvertent disclosure by lay health workers.

^d^2/11 Lost to Follow Up women stopped ART after their relationship ended because they did not want to take ART on their own. 2/11 LTFU women were discouraged from continuing ART by their male partner because the male partner feared ART side effects for both individuals. 3/11 Lost to Follow Up women in serodiscordant relationships stopped ART after being separated from their partner due to HIV disclosure by the woman.

Table 4 presents quotes from study participants illustrating primary reasons for non-disclosure between partners.

**Table 4:** Main reasons for non-disclosure within couples.

**Fear of rejection and end of relationship**
*Most [women who do not disclose to their partner] are afraid that their husband will leave them*. *This is the most common reason that women* […] *give for not disclosing their status to their husband and […] it took me a month to tell my husband because I was afraid he would leave me*. (Adult woman, 27-year-old, 104-107-11-001)
*If I tell my husband then he will chase me and the marriage won’t be there*, *there are several people that I have seen who explained to the husband and he left* (Adult woman, 33-year-old, 103-106-11-003)
*Most people are scared that if I tell my spouse*, *they might end up leaving me so they would rather stay quiet waiting for the spouse to go and get tested when they feel like it*. (Adult woman, 29-year-old, 103-105-11-001
*Men are short tempered and women are scared that telling them that they have the virus might mean the end of the marriage*. (Adult woman, 26-year-old 106-110-11-002)
*I did not know what to say to my wife and how to say it at that time*. *I was afraid she was going to deny my proposal and I thought it was better to tell her after we get married*. (Male partner, 36-year-old, 108-113-33-005)
*When I explained to my husband he told me that I am a whore so we have to end the marriage up to the extent that he asked for a transfer [ART services received at another hospital]*. (LTFU, 27-year-old 102-103-22-001
**Gender based dynamics and intimate partner violence**
*Gender based violence towards women who disclose their HIV status make it hard for women to open up about their status*. (Adult woman, 38-year-old, 106-110-11-004)
*[Women who do not disclose to their partner] are afraid that [the partner] will beat them up or they can be killed or the marriage would end*. (Adult woman, 36-year-old, 107-112-11-003)
*This is a threat to women*, *it prevents them from opening up*: […] *women hide their status depending on the behavior of their spouse*. […] *Because of the character of the husband most women don’t know how to go about telling the husband who is difficult*. (Adult woman, 27-year-old, 103-104-11-003
*Some men are terrifying*. […] *they search where you keep your medicine and throw them away*. (Adult woman, 27-year-old, 104-107-11-001

The most prominent reason for non-disclosure to partners was women’s fear of rejection by their male partner; some men also expressed not disclosing to their partner because of the fear of being rejected; mothers often spontaneously associated the disclosure of their positive HIV status to their partner with the end of their relationship and with being discriminated and/or being accused of promiscuity and extra-marital sexual activity. Data also showed that gender-based dynamics and fear of the partner’s reaction were key reasons for mothers experiencing difficulties in disclosing. Mothers expressed feeling at risk and vulnerable when disclosing to their partners. These difficulties were often linked to poor ART adherence and some mothers said that hiding medication from their partner was impossible.

In contrast, within couples who had mutually disclosed, qualitative data (Table 5) indicate that they had developed mutual support strategies for adherence and retention in care. In some couples, partners helped each other by reminding their spouse to take medication at specific times or by taking ART together. Some mothers also stated that not having to hide medication, as a result of having disclosed their ART status to their partner, was helpful to continue taking ART.

**Table 5:** Couple support strategies amongst couples who disclosed to each other.

*When my husband is around and it is time to take our medication*, *I remind him that it is time so all of us*, *him*, *me and our children take the medication at the same time and then we go to sleep*. (Adult woman, 35-year-old, 102-103-11-001)
*You encourage each other because you are able to remind each other about the time to take medication* […] *but if you hide it becomes hard for you to leave and take medicine*. (Adult woman, 33-year-old, 103-105-11-003)
*You encourage each other in your house [to take ART]*. (Adult woman, 31-year-old, 103-106-11-001)
*We have to remind each other to take the drugs which is helping keeping us healthy*. (Adult woman, 22-year-old, 106-110-11-001)
*When you are taking the medication you do not take it in fear because you know that you have told your husband*. (Adult woman, 37-year-old, 104-107-11-003)

## Discussion

Within a cohort of Malawian women who were 4–26 weeks postpartum, we present detailed associations between partner HIV status, disclosure patterns, and PMTCT utilization and outcomes. While maternal disclosure alone was high in this cohort, full disclosure between partners occurred in less than three quarters of couples. Non-disclosure of both partners was a strong predictor of no ART uptake, suboptimal treatment adherence, and increased MTCT. Qualitative data illustrated reasons for difficulties in treatment adherence for mothers who did not disclose to their partner, some of whom became LTFU. Mothers experienced disclosure as an enabling factor for continuing to take ART as a result of increased partner support whilst others feared or experienced active discouragement by their partner to continue ART after disclosing.

In the same nationally-representative cohort we recently described that the Option B+ PMTCT strategy in Malawi has led to high uptake and adherence, but that missing steps in the PMTCT cascade of care increases the risk of MTCT [19]. Further reduction of MTCT in the Option B+ program may depend on recognizing and understanding socio-behavioral factors such as between-partner disclosure and the impact this can have on a woman’s ability to fully engage in the PMTCT cascade of care.

Here we demonstrate that over 90% of mothers reported that they had disclosed their HIV status to their partner. This is much higher than the <30% in an urban cohort in Malawi prior to Option B+ [20] and the 70–77% reported in other regional studies [14, 15]. It is plausible that the 2011 change in Malawi’s Integrated HIV/ART Guidelines has facilitated support for maternal disclosure and male involvement in PMTCT by encouraging reorganization and integration of health services to include family-centered care and decentralization of ART services [21]. Furthermore, the literature reports several ongoing studies and programs within Malawi that encourage disclosure and male involvement, perhaps signaling a cultural shift in engagement of men in antenatal care and PMTCT services, including mutual disclosure of HIV status [6, 10, 22–24].

While maternal disclosure was high in this study, we report that the level of full between-partner disclosure was moderate and that being in a relationship in which neither partner disclosed their HIV status was a strong predictor of missing maternal ART uptake, suboptimal treatment adherence, and increased MTCT. Additionally, mothers without a partner, or mothers in relationships where only one partner disclosed were also at risk of suboptimal treatment adherence. We found no other studies describing disclosure patterns for women in PMTCT programs in detail, and as illustrated by our qualitative data, these findings may represent relationship dynamics driven by fear or perceived stigma, which may limit open communication and ultimately hinder women to achieve optimal PMTCT utilization [25, 26]. The dynamics of disclosure within couples in Option B+ PMTCT care need to be contextualized within gender and cultural norms that present fewer opportunities for women to make autonomous decisions in relation to day-to-day health-seeking behaviors [27]. Beyond the act of disclosing, living in a predominantly patriarchal society translates into fewer opportunities for women’s empowerment and there are currently limited specialized resources for women seeking support to deal with HIV disclosure within couples.

While we cannot comment specifically on the lack of male involvement in HIV care among couples in whom neither of the partners disclosed, it is likely to be limited. This small but important group of mostly younger women in relationships without any disclosure between partners is likely to be at highest risk of MTCT.

Finally, we did not find an association between the partner’s HIV status and PMTCT utilization and outcomes among couples who had mutually disclosed. Mothers who reported discordant disclosure between partners were equally likely to attain and adhere to maternal ART and had similar outcomes as mothers who were in relationships with concordant HIV-positive status. There are no comparable studies in the literature to date, and our results indicate that a relationship that allows open communication and trust between partners may be more impactful on MTCT than the partner’s HIV status.

The strength of this study was its large sample and the mixed-methods approach which allowed contextualization of the findings of PMCT utilization in relation to disclosure patterns. However, quantitative analysis is limited by the fact that partner disclosure status, ART uptake and adherence, and infant care were based on self-report by the mother only. Qualitative analysis is limited by the lack of available data about specific reasons for non-disclosure by male partners. Additionally, this analysis controlled for known confounders however there could be other characteristics of non-disclosing couples, other than disclosure patterns, that led to poorer outcomes. The cross-sectional nature of this study does not directly allow for causal inference. It is possible that participants who were adherent were also more likely to disclose.

Lastly, as some of the numbers in the subgroup analyses were small, the reported confidence intervals are relatively wide and need to be interpreted with care.

## Conclusion

Given the strong link we found between inter-partner non-disclosure and uptake of and adherence to ART and ultimately MTCT outcomes in Malawi, a clearer understanding of the causes and consequences of inter-partner disclosure in the context of Option B+ and “Test and Start” in Malawi is needed. HCWs in PMTCT programs should be trained to monitor disclosure, identify reasons why individual women do not disclose and then to provide tailored measures that facilitate disclosure (such as couple disclosure counseling or addressing gender-based violence in couples), in order to further improve the effectiveness of PMTCT services.

## Supporting information

S1 File Questionnaires and interview guides in English and Chichewa. (DOCX)

S2 File NEMAPP partner disclosure dataset. (XLSX)
