# Supplementary material for: Impact of inter-partner HIV disclosure patterns in Malawi’s PMTCT program: A mixed-method study
Source: PLoS One. 2019 Jul 26;14(7):e0219967. doi: 10.1371/journal.pone.0219967 (PMC6660128; doi:10.1371/journal.pone.0219967)
Supplement: S1 File — (DOCX) [file pone.0219967.s001.docx]

# DATA COLLECTION TOOLS

## English Versions

### Interview Guide for Women

| **Interview Guide: WOMEN**  NEMAPP Qualitative Study |
| --- |
| **Interview #: 01 / 02 / 03 / 04**  **NEMAPP Qualitative #:**  **District:**  **Facility/Site:**  **Date of study enrolment in NEMAPP main study: (day/month/year)**  **Main NEMAPP study number:**  **Age of Respondent:**  **Marital Status:**  1= Married  2=Widowed  3=Separated  4=Never married  5=Other (specify)________________________________  **Qualification**  1= None  2=Junior primary  3= Senior primary  4=Junior secondary  5=Senior secondary  6=University degree  7=Other certificates and diplomas (specify)__________________________  8=Other (specify)____________________________________________________  **Ethnicity**  1 = Yao  2= Lomwe  3= Chewa  4= Tumbuka  5= Ngoni  6=Sena  7= Other (specify)________________________________  **Religious background:**  1 = Christianty chikhilisitu  2= Islam chisilamu  3=Traditional chikhalidwe  4 = None or Other (Specify) ___________________________  **Number of children:**  **Currently Pregnant:**  1= Yes  2= No  **Curently Breastfeeding: If yes, since:**  1=Yes  2= No  **Interviewer/Moderator:**  **DD/MM/YYYY**  **HRS**  **HRS**  **Date: Start time: End time:** |
| **Instructions for Interviewer:**   - Follow the informed consent procedures, including asking for permission to record the interviews. Give ample time for the respondent to make up her mind. The respondent should be comfortable to discuss with you for at least 60 minutes (reschedule if this is not possible). - You are responsible to ensure privacy (including audio-privacy) during this interview. - This is a guide for in-depth interviewing (not a survey): probe as much as possible into the response from the respondent for clarification and explore each of the key themes in depth. The interview should flow like a discussion rather than a question-answer session. - From this point onwards, the interview should be recorded, with permission from the respondent. - Makes notes of your observations directly at the end of the form, on the same day of the interview. |
| 1. *[Set of introductory questions*:*]* To start our discussion today, could you please introduce yourself? Could you describe your family?   *[Probe]: Who lives in your household? How long have you lived in this area? What type of work do you do? What do you enjoy doing when you have some fee time?*  *As I mentioned earlier [refer to the informed consent discussion], I am now going to ask you a few questions related to HIV. I hope this is ok with you and that you feel comfortable about this interview. Just to remind you that if you feel uncomfortable about any of these questions, you don’t need to answer and we can move on to the next.*   1. How long have you attended services for HIV at this health facility [or site]?   *[Probe]: Is this the first time you attend HIV services?*  *Did you attend another health facility previously? If yes, which one and for how long?*  *Why did you choose to come to this specific health facility?*   1. Could you please describe the type of support that you currently receive in relation to HIV?   *[Probe]: Do you receive additional support from family members, partners, health care workers, expert patients, friends, or others?*   1. Let's go back to when you were tested for HIV, if that’s ok. Could you tell me more about the time when you tested for HIV?   *[Probe]: Why did you take a test? Were you encouraged to do so? Did you choose to test? Did you test with someone else? Did you test with your partner?*  *How did you react when you were told about test results?*  *Who did you share the results with?*  *What support did you have at that time, if any?*   1. Were you asked to start taking treatment (ART) for HIV?   *[Probe]: When were you asked to start taking treatment (ART) for HIV?*  *What was explain to you at that time?*  *How long will you need to take this treatment for?*  *How did you feel about taking this treatment when you started?*  *Could you choose to start the treatment? [Probe: What decisions did you make at the time, what influenced your decisions?]*   1. Could you describe what happened after you were asked to take HIV treatment (ART)?   *[Probe]: Who have you discussed the HIV treatment with?*  *How long after the HIV test did you start taking the treatment for he first time?*  *Did you continue to take the treatment? If not, could you describe what happened [Probe into the reasons for not continuing treatment, and when treatment was stopped, started again, or discontinued]?*   1. Do you take treatment regularly?   *[Probe]: How do you feel about the HIV treatment?*  *If you don’t take your treatment sometimes, what are the reasons?*   1. Did you talk to you partner about the HIV test?   *[Probe]: How about your treatment for HIV? Did you discuss it with your partner?*  *Has your partner ever been tested for HIV? If yes, did he share the results of the test with you?*  *Do you know if your partner is taking HIV treatment at the moment?*  *Have you ever shared your HIV treatment with your partner or someone else? [If yes, probe: why, whom with, describe how this took place, was it just once or do you share treatment regularly?]*  *Who else have you shared or discussed your HIV status with? [Probe: when did you disclose for each of these individuals? what was the process, where did this happen, how did they react?].*   1. What advice did you receive in relation to breastfeeding?   *[Probe]: Since you strated to take ART, have you changed anything in relation to breastfeeding your baby?*  *[Probe further to understand if the perception and/or practice of breastfeeding has changed since starting ART]*   1. Would you like to get pregnant again in the future?   *[Probe]: Did taking ART made you feel different about getting pregnant again in the future?*  *[Probe further to understand if ART has impacted perception of safer or riskier pregnancy]*   1. In your personal opinion, are there specific things that could be done to improve the process of testing for HIV and starting HIV treatment?   *[Probe]: Where you comfortable to start HIV treatment quickly after the test?*  *In your opinion, what type of support should ideally be provided for mothers and their families as part of HIV care?* |
| **Additional Comments**  Before we close this interview, please tell us if there is anything you would like to add in relation to the topics that we discussed? *[Describe]* |
| **Instructions for interviewer**:   - Thank the respondent for their time and remind her that we would like to interview her again in 6 months, if she agrees. - After the interview (on the same day) note down here any observations that help contextualize the data collected (i.e. location, flow of the discussion, interruptions, flow of the interview, emotions, hesitation, description of the respondent, etc.) |

### Interview Guide for LTFU women

| **Interview Guide: LTFU WOMEN**  NEMAPP Qualitative Study |
| --- |
| **NEMAPP Qualitative #:**  **Interview #: 01 / 02 / 03 / 04**  **District:**  **Facility/Site:**  **Date of study enrolment in NEMAPP main study: (day/month/year)**  **Main NEMAPP study number:**  **Age of Respondent:**  **Marital Status:**  1= Married  2=Widowed  3=Separated  4=Never married  5=Other (specify)________________________________  **Qualification**  1= None  2=Junior primary  3= Senior primary  4=Junior secondary  5=Senior secondary  6=University degree  7=Other certificates and diplomas (specify)__________________________  8=Other (specify)____________________________________________________  **Ethnicity**  1 = Yao  2= Lomwe  3= Chewa  4= Tumbuka  5= Ngoni  6=Sena  7= Other (specify)________________________________  **Religious background:**  1 = Christianty chikhilisitu  2= Islam chisilamu  3=Traditional chikhalidwe  4 = None or Other (Specify) ___________________________  **Number of children:**  **Currently Pregnant:**  1= Yes  2= No  **Curently Breastfeeding: If yes, since:**  1=Yes  2= No  **Interviewer:**  **DD/MM/YYYY**  **HRS**  **HRS**  **Date: Start time: End time:** |
| **Instructions for Interviewer:**   - Follow the informed consent procedures, including asking for permission to record the interviews. Give ample time for the respondent to make up her mind. The respondent should be comfortable to discuss with you for at least 60 minutes (reschedule if this is not possible). - You are responsible to ensure privacy (including audio-privacy) during this interview. - This is a guide for in-depth interviewing (not a survey): probe as much as possible into the response from the respondent for clarification and explore each of the key themes in depth. The interview should flow like a discussion rather than a question-answer session. - From this point onwards, the interview should be recorded, with permission from the respondent. - Makes notes of your observations directly at the end of the form, on the same day of the interview. |
| 1. *[Set of introductory questions*:*]* To start our discussion today, could you please introduce yourself? Could you describe your family?   *[Probe]: Who lives in your household? How long have you lived in this area? What type of work do you do? What do you enjoy doing when you have some fee time?*  *As I mentioned earlier [refer to the informed consent discussion], I am now going to ask you a few questions related to HIV. I hope this is ok with you and that you feel comfortable about this interview. Just to remind you that if you feel uncomfortable about any of these questions, you don’t need to answer and we can move on to the next.*   1. We noticed that you have not continued to attend HIV services. What were the reasons why you decided not to continue to attend HIV services?   *[Probe]: Could you describe how you felt about the HIV services at the facility which you visited?*  *Could you describe how you felt about the HIV treamtment?*   1. How long have you attended services for HIV, before you stopped visiting the health facility?   *[Probe]: Was this the first time you attended HIV services?*  *Did you attend another health facility previously? If yes, which one and for how long?*  *Why did you choose to come to this specific health facility?*   1. Could you please describe the type of support that you currently receive in relation to HIV?   *[Probe]: Do you receive additional support from family members, partners, health care workers, expert patients, friends, or others?*   1. Let's go back to when you were tested for HIV, if that’s ok. Could you tell me more about the time when you tested for HIV?   *[Probe]: Why did you take a test? Were you encouraged to do so? Did you choose to test? Did you test with someone else? Did you test with your partner?*  *How did you react when you were told about test results?*  *Who did you share the results with?*  *What support did you have at that time, if any?*   1. Were you asked to start taking treatment (ART) for HIV?   *[Probe]: When were you asked to start taking treatment (ART) for HIV?*  *What was explain to you at that time?*  *How long would you need to take this treatment for, if you take it?*  *How did you feel about taking this treatment when you started the first time?*  *If you started to take ART, did you choose to start the treatment? [Probe: What decisions did you make at the time, what influenced your decisions?]*   1. Could you describe what happened after you were asked to take HIV treatment (ART)?   *[Probe]: Who have you discussed the HIV treatment with?*  *How long after the HIV test did you start taking the treatment fot he first time?*  *Did you continue to take the treatment? If not, could you describe what happened*  *[Probe into the reasons for not continuing treatment, and when treatment was stopped, started again, or discontinued]?*   1. We noticed that you didn’t go back to the health facility lately. Did you continue to take the treatment without going back to the health facility, or did you stop?   *[Probe]: If you continued to take ART, where did you find it?*  *How do/did you feel about the HIV treatment?*  *If you don’t take your treatment at the moment, what are the reasons?*   1. Did you talk to you partner about the HIV test?   *[Probe]: How about your treatment for HIV? Did you discuss it with your partner?*  *Has your partner ever been tested for HIV? If yes, did he share the results of the test with you?*  *Do you know if your partner is taking HIV treatment at the moment?*  *Have you ever shared your HIV treatment with your partner or someone else? [If yes, probe: why, whom with, describe how this took place, was it just once or do you share treatment regularly?]*  *Who else have you shared or discussed your HIV status with? [Probe: when did you disclose for each of these individuals? what was the process, where did this happen, how did they react?].*   1. What advice did you receive in relation to breastfeeding?   *[Probe]: Since you strated to take ART, have you changed anything in relation to breastfeeding your baby?*  *[Probe further to understand if the perception and/or practice of breastfeeding has changed since starting ART]*   1. Would you like to get pregnant again in the future?   *[Probe]: Did taking ART made you feel different about getting pregnant again in the future?*  *[Probe further to understand if ART has impacted perception of safer or riskier pregnancy]*   1. In your personal opinion, are there specific things that could be done to improve the process of testing for HIV and starting HIV treatment?   *[Probe]: Where you comfortable to start HIV treatment quickly after the test?*  *In your opinion, what type of support should ideally be provided for mothers and their families as part of HIV care?* |
| **Additional Comments**  Before we close this interview, please tell us if there is anything you would like to add in relation to the topics that we discussed? *[Describe]* |
| **Instructions for interviewer**:   - Thank the respondent for their time and remind her that we would like to interview her again in 6 months, if she agrees. - After the interview (on the same day) note down here any observations that help contextualize the data collected (i.e. location, flow of the discussion, interruptions, flow of the interview, emotions, hesitation, description of the respondent, etc.) |

### Focus group discussion guide for Health care workers

| **Focus Group Discussion Guide: Health Care Workers (involved in providing B+)**  NEMAPP Qualitative Study | | | | |
| --- | --- | --- | --- | --- |
| **FGD #: District:**  **Facility:**  **AM/PM**  **AM/PM**  **Date: Start time: End time:** | | | | |
|  | **Age** | **Gender** | **Education** | **Cadre (Nurse, HSA, Medical Officer, etc.)** |
| 1 |  |  |  |  |
| 2 |  |  |  |  |
| 3 |  |  |  |  |
| 4 |  |  |  |  |
| 5 |  |  |  |  |
| 6 |  |  |  |  |
| **Education:**  1= None, 2=Junior primary, 3= Senior primary, 4=Junior secondary, 5=Senior secondary, 6=University degree, 7=Other certificates and diplomas (specify), 8=Other (specify)  **FGD Moderator:** | | | | |
| **Instructions for FGD Moderator:**   - This guide should help you to initiate and moderate the discussion, which should flow like a normal discussion rather than a question-answers session - The guide is designed for a group of 4-6 health care workers, to be conducted once in each health facility. Ideally, the group should include both men and women. - Participants must be aware that focus group discussions will take around 60 minutes (reschedule if this is not possible) - Ask another member of the reseach team to take notes about interactions, group dynamics, and the content of discussions. - Ensure privacy (including audio-privacy) for the discussion. Participants are likely to know each other already. Establish some ground rule about the confidentiality of what is discussed within the group. | | | | |
| We would like to discuss how PMTCT care is organised and listen to your observations about the day to day activities in this health centre   1. What is your perception of the PMTCT care being provided, what is good and what could be improved? 2. How would you describe the experience of women attending care for PMTCT in this facility? 3. In your opinion, what is done or could be done to include male partners in these services? 4. Are there any services that you think are missing? Any other types of support needed for women and their families? 5. How do you feel about the processes involved in starting patients on ART?   In your opinion, what could be improved in the processes involved in starting treatment?     1. Could you describe how you perceive Option B+?   What does this programme change on issues related to HIV treatment, testing, breastfeeding and male partner involvement?   1. In your opinion, why do you think some women are lost to follow up?   What are the reasons for LTFU, in your experience? What can the PMTCT service do to lower the rate of LTFU?   1. What would be the best experience of care in your opinion, i.e. the best experience that the health service could provide for mothers and/or pregnant women in this context? What is the top health issue that your health facility is focusing on? **(describe)** | | | | |
| **Instructions for FGD moderator:**   - Thank participants for their time and explain that we would like to have another focus group in about 6 months. - On the same day, record any additional observations directly after the FGD – note anything relevant to group dynamics, power relations, interruptions, and any other important elements to help contextualise the content of the discussions. | | | | |

### Interview Guide for Male Partners

| **Interview Guide: Male Partners**  NEMAPP Qualitative Study |
| --- |
| **NEMAPP Qualitative #:**  **Interview #: 01 / 02 / 03 / 04**  **District:**  **Facility/Site:**  **Date of study enrolment in NEMAPP main study: (day/month/year)**  **Main NEMAPP study number:**  **Age of Respondent:**  **Marital Status:**  1= Married  2=Widowed  3=Separated  4=Never married  5=Other (specify)________________________________  **Qualification**  1= None  2=Junior primary  3= Senior primary  4=Junior secondary  5=Senior secondary  6=University degree  7=Other certificates and diplomas (specify)__________________________  8=Other (specify)____________________________________________________  **Ethnicity**  1 = Yao  2= Lomwe  3= Chewa  4= Tumbuka  5= Ngoni  6=Sena  7= Other (specify)________________________________  **Religious background:**  1 = Christianty chikhilisitu  2= Islam chisilamu  3=Traditional chikhalidwe  4 = None or Other (Specify) ___________________________  **Number of children:**  **Partner currently Pregnant:**  1= Yes  2= No  **Patner Breastfeeding: If yes, since:**  1=Yes  2= No  **Interviewer/Moderator:**  **HRS**  **HRS**  **Date: Start time: End time:** |
| **Instructions for Interviewer:**   - Follow the informed consent process, including asking for permission to record the interviews. Give ample time for the respondent to make up his mind. The respondent should be comfortable to discuss with you for at least 60 minutes (reschedule if this is not possible). - You are responsible to ensure privacy (including audio-privacy) during this interview. - This is a guide for in-depth interviewing (not a survey): probe as much as possible into the response from the respondent for clarification and explore each of the key themes in depth. The interview should flow like a discussion rather than a question-answer session. - From this point onwards, the interview should be recorded, it will be fully transcribed later. - Note your observations directly at the end of the form, on the same day of the interview. |
| 1. Do you currently attend any services at the health facility which you partner attends? ***[Probe which services].*** 2. Could you describe to me if you ever had an HIV test and any kind of support that you have received before or after testing? 3. Could you describe the support that your partner currently receives in relation to HIV? ***[Probe for support for family members, partners, health care workers, expert patients, friends, other].*** 4. Is the support that she currently receive for HIV, including HIV treatment, different from other health services? ***[How? Please explain].***   5. Do you feel that the treatment that your partner receives may impact on you? *[Probe: how?]*  6. Do you receive any similar support currently? ***[Probe]*** |
| ***7. [If ever tested for HIV]:*** Could you tell me more about the time when you tested for HIV? *[Probe: Why did you take a test? How did you react when you were told about test results? Who did you share the results with? What support did you have at that time?]*  ***8. [If ever tested and received positive HIV results]:*** When were you asked to start taking treatment (ART) for HIV? *[Probe: How long after the test did you start taking treatment for HIV?]*   1. ***[If taking HIV treatment]:*** Tell me about your experience from the moment you were tested for HIV and started treatment, up to now. 2. ***[If taking HIV treatment]:*** Do you take treatment regularly? *[Probe into any challenges in taking treatment]* 3. Are you aware of any specific challenges that your partner has experienced in taking HIV treatment? *[Probe].* 4. Would you like to have more children in the future? *[Probe: Could you describe the reasons for wanting/not wanting more children in the future? Do you think that your partner taking HIV treatment has anything to do with having more children? Why?].* 5. In your personal opinion, are there specific things that could be done to improve the process of testing for HIV and start treatment? 6. Have you encountered men who did not want to test or take HIV treatment? *[Probe: Describe their reasons]*   15. In your opinion, what type of support should ideally be provided for mothers and their families as part of HIV care? |
| 1. ***[If ever tested for HIV]:*** Who have you shared your HIV status with? *[Probe: when did you disclose for each of these individuals?].*   ***17. [If ever tested for HIV]:*** Tell us about how you disclosed to *[individuals cited above]*? *[Probe: what was the process, where did this happen, how did they react?]*  ***18.. [If ever tested for HIV***]: Did you share the results of your test with your partner? *[If not, ask why/ if yes, ask to describe the process].*   1. Do you think it is important for someone to disclose his or her HIV status to others? Why? *[Probe any challenges cited].* |
| Before we close this interview, please tell us if there is anything you would like to add in relation to the topics that we discussed? *[Describe]* |
| **Instructions for interviewer**:   - Thank the respondent for their time and explain that we would like to interview them again in 6 months. - After the interview (on the same day) note down here any observations that help contextualize the data collected (i.e. location, flow of the discussion, interruptions, flow of the interview, emotions, hesitation, description of the respondent, etc.) |

### Interview Guide for Community Leaders

| **Interview Guide: Community Leaders**  NEMAPP Qualitative Study |
| --- |
| **NEMAPP Qualitative #:**  **Interview #: 01 / 02 / 03 / 04**  **District:**  **Facility/Site:**  **Age of Respondent:**  **Marital Status:**  1= Married  2=Widowed  3=Separated  4=Never married  5=Other (specify)________________________________  **Qualification**  1= None  2=Junior primary  3= Senior primary  4=Junior secondary  5=Senior secondary  6=University degree  7=Other certificates and diplomas (specify)__________________________  8=Other (specify)____________________________________________________  **Ethnicity**  1 = Yao  2= Lomwe  3= Chewa  4= Tumbuka  5= Ngoni  6=Sena  7= Other (specify)________________________________  **Religious background:**  1 = Christianty chikhilisitu  2= Islam chisilamu  3=Traditional chikhalidwe  4 = None or Other (Specify) ___________________________  **Male/Female:**  **Interviewer/Moderator:**    **HRS**  **HRS**  **Date: Start time: End time:** |
| **Instructions for Interviewer:**   - Follow the informed consent process, including asking for permission to record the interviews. Give ample time for the respondent to make up his/her mind. The respondent should be comfortable to discuss with you for at least 60 minutes (reschedule if this is not possible). - You are responsible to ensure privacy (including audio-privacy) during this interview. - This is a guide for in-depth interviewing (not a survey): probe as much as possible into the response from the respondent for clarification and explore each of the key themes in depth. The interview should flow like a discussion rather than a question-answer session. - From this point onwards, the interview should be recorded, it will be fully transcribed later. - Note your observations directly at the end of the form, on the same day of the interview. |
| 1. What is your perception of the PMTCT care provided currently at the health facility in this community?   *[Probe]:* What is good? What could be improved?   1. Could you describe the experience of women attending care for PMTCT at the health facility accessed by your community?   *[Probe]:* Do the women that you know and who have access PMTCT services reported any specific views or concerns?   1. In your opinion, what is done or could be done to include male partners in these services? 2. Are there any key services that you think are missing? Are there any other types of support needed for women and their families? 3. How do you feel about the processes involved in starting patients on HIV treatment, specifically for women attending PMTCT services?   *[Probe]:* In your opinion, what could be improved in the processes involved in starting treatment?   1. Do you know the term: ‘Option B+’? If yes, could you describe how you perceive Option B+?   *[Probe]:* Fom your point of view, how does this programme impact on issues related to HIV treatment, testing, breastfeeding and male partner involvement?   1. Do you think that having access to lifelong ART treatment could impact on breastfeeding in this community?   *[Probe]:* How does it impact breastfeeling and whom does it impact most?   1. Do you think that having access to lifelong ART treatment could impact a woman’s intention to get pregnant again in this community?   *[Probe]:* How does it impact future pregnancy intentions and whom does it impact most?   1. In your opinion, why do you think some women do not come back to pick up treatment or for scheduled visits to the health facility?   *[Probe]:* What are the reasons for women stopping treatment, from your perspective? What can PMTCT services do to encourage women to come back to the health facilities for HIV care and treatment?   1. What would be the best experience of care in your opinion, that is the best experience that the health service could provide for mothers and/or pregnant women in this community?   *[Probe]:* Could you give us an example of the type of PMTCT services that would be needed specifically in this community?   1. In your community, is privacy and confidentiality in relation to HIV a concern? Is HIV talked about much?   *[Probe]:* What are the key concerns and how are they addressed? Do you think that stigma in relation to people living with HIV has changed in the past few months or years? If yes, please describe how it has changed. If no, please describe why you think it hasn’t changed.   1. From your own perspective, are there any specific barriers that would prevent some women who are living with HIV from accessing PMTCT care?   *[Probe]:* Could you describe these barriers? How important are they in this community? What could improve the access to HIV testing and treatment services?   1. Are there any factors that have helped some women who are living with HIV to acess care and treatment in this community?   *[Probe]:* Could you describe these factors? How have they benefited women in this community? |
| Before we close this interview, please tell us if there is anything you would like to add in relation to the topics that we discussed? *[Describe]* |
| **Instructions for interviewer**:   - Thank the respondent for their time and explain that we would like to interview them again in 6 months. - After the interview (on the same day) note down here any observations that help contextualize the data collected (i.e. location, flow of the discussion, interruptions, flow of the interview, emotions, hesitation, description of the respondent, etc.) |

### Interview Guide for PMTCT Focal persons

| **Interview Guide: PMTCT Focal Persons**  NEMAPP Qualitative Study |
| --- |
| **NEMAPP Qualitative #:**  **Interview #: 01 / 02 / 03 / 04**  **District:**  **Facility/Site:**  **Age of Respondent:**  **Marital Status:**  1= Married  2=Widowed  3=Separated  4=Never married  5=Other (specify)________________________________  **Qualification**  1= None  2=Junior primary  3= Senior primary  4=Junior secondary  5=Senior secondary  6=University degree  7=Other certificates and diplomas (specify)__________________________  8=Other (specify)____________________________________________________  **Ethnicity**  1 = Yao  2= Lomwe  3= Chewa  4= Tumbuka  5= Ngoni  6=Sena  7= Other (specify)________________________________  **Religious background:**  1 = Christianty chikhilisitu  2= Islam chisilamu  3=Traditional chikhalidwe  4 = None or Other (Specify) ___________________________  **Male/Female:**  **Interviewer/Moderator:**    **HRS**  **HRS**  **Date: Start time: End time:** |
| **Instructions for Interviewer:**   - Follow the informed consent process, including asking for permission to record the interviews. Give ample time for the respondent to make up his/her mind. The respondent should be comfortable to discuss with you for at least 60 minutes (reschedule if this is not possible). - You are responsible to ensure privacy (including audio-privacy) during this interview. - This is a guide for in-depth interviewing (not a survey): probe as much as possible into the response from the respondent for clarification and explore each of the key themes in depth. The interview should flow like a discussion rather than a question-answer session. - From this point onwards, the interview should be recorded, it will be fully transcribed later. - Note your observations directly at the end of the form, on the same day of the interview. |
| 1. What is your perception of the PMTCT care provided currently at the health facility in this district?   *[Probe]:* What is good? What could be improved?   1. Could you describe the experience of women attending care for PMTCT at the health facility accessed in this district?   *[Probe]:* Do women who have access PMTCT services reported any specific views or concerns?   1. In your opinion, what is done or could be done to include male partners in PMTCT services? 2. Are there any key services that you think are missing? Are there any other types of support needed for women and their families? 3. How do you feel about the processes involved in starting patients on HIV treatment, specifically for women attending PMTCT services?   *[Probe]:* In your opinion, what could be improved in the processes involved in starting treatment?   1. Could you describe how you perceive Option B+?   *[Probe]:* Fom your point of view, how does this programme impact on issues related to HIV treatment, testing, breastfeeding and male partner involvement?   1. Do you think that having access to lifelong ART treatment could impact on breastfeeding in this district?   *[Probe]:* How does it impact breastfeeling and whom does it impact most?   1. Do you think that having access to lifelong ART treatment could impact a woman’s intention to get pregnant again in this district?   *[Probe]:* How does it impact future pregnancy intentions and whom does it impact most?   1. In your opinion, why do you think some women do not come back to pick up treatment or for scheduled visits to the health facilities located in this disctrict?   *[Probe]:* What are the reasons for women stopping treatment, from your perspective? What can PMTCT services do to encourage women to come back to the health facilities for HIV care and treatment?   1. What would be the best experience of care in your opinion, that is the best experience that the health service could provide for mothers and/or pregnant women in this community?   *[Probe]:* Could you give us an example of the type of PMTCT services that would be needed specifically in this district?   1. In this district, is privacy and confidentiality in relation to HIV a concern? Is HIV talked about much?   *[Probe]:* What are the key concerns and how are they addressed? Do you think that stigma in relation to people living with HIV has changed in the past few months or years? If yes, please describe how it has changed. If no, please describe why you think it hasn’t changed.   1. From your own perspective, are there any specific barriers that would prevent some women who are living with HIV from accessing PMTCT care?   *[Probe]:* Could you describe these barriers? How important are they in this district? What could improve the access to HIV testing and treatment services?   1. Are there any factors that have helped some women who are living with HIV to acess care and treatment in this district?   *[Probe]:* Could you describe these factors? How have they benefited women in this district? |
| Before we close this interview, please tell us if there is anything you would like to add in relation to the topics that we discussed? *[Describe]* |
| **Instructions for interviewer**:   - Thank the respondent for their time and explain that we would like to interview them again in 6 months. - After the interview (on the same day) note down here any observations that help contextualize the data collected (i.e. location, flow of the discussion, interruptions, flow of the interview, emotions, hesitation, description of the respondent, etc.) |

## Chichewa Versions

### Interview Guide for Women (Chichewa)

| **Dongosolo lofunsira mafunso: AMAYI OYEMBEKEZERA NDI OYAMWITSA**  Kafukufuku wa NEMAPP |
| --- |
| **Nambala ya**  **macheza: Boma:**  **Chipatala/Malo:**  **Tsiku lolowera mukafukufuku: (Tsiku) (Mwezi)**  **Nambala yamu kafukufuku**  **wa NEMAPP:**  **Zaka za ofunsidwa mafunso:**  **Ndiokwatiwa kapena**  **osakwatiwa:**  1= Okwatiwa  2= Nkazi wa mamasiye  3= Anasiyana  4= Sanakwatiwepo  5= China (longosoloni)________________________________  **Maphunziro**  1= Sanaphunzire  2= Makalasi am’munsi ku pulaimale  3= Makalasi a m’mwamba ku pulaimale  4= Makalasi a m’munsi ku sekondale  5= Makalasi a m’mwamba kusekondale  6= Adafika pa ukachenjede  7= Satifiketi kapena Dipuloma (longosolani)__________________________  8= China (longosolani)____________________________________________________  **Mtundu**  1= Muyao  2= Mulomwe  3= Mchewa  4= Mtumbuka  5= Mngoni  6= Msena  7= Wina (longosolani)________________________________  **Chipembedzo:**  1= Chikhilisitu  2= Chisilamu  3= Chikhalidwe  4 = Palibe kapena china (longosolani) ___________________________  **Ali ndi ana angati:**  **Ndiwoyembekezera?**  1= Inde  2= Ayi  **Akuyamwitsa? If yes Ngati akuyamwitsa, ayamba liti?**  1= Inde  2= Ayi  **Ofunsa mafunso/Otsogolera:**  **TSIKU/MWEZI.CHAKA**  **HRS**  **HRS**  **Tsiku: Nthawi yoyambira: Nthawi yomalizira:** |
| **Malangizo kwa ofunsa mafunso:**   - Tsalirani malamulo otengela chilolezo kuphatikizapo chilolezo chotenga macheza ndi chintapa mau. Mupatseni yemwe mukumufunsa mafunsoyo nthawi yokwanira yoti apangire chiganizo. Yemwe mukucheza nayeyo akhale omasuka kucheza nanu nthawi yopitilira ola limodzi (Ngati sangathe, pezani tsiku lina). - Mukuyenera kuwonetsetsa kuti chinsinsi chisungika (kuphatikizapo chinsinsi kuti zomwe zikulankhulidwa zisamveke) pa nthawi yomwe mukucheza. - Ili ndi dongosolo logwiritsidwa ntchito pa zokambirana zozama (osati pongofufuza pang’ono): mukuyenera kufunsa momwe mungathere pa zomwe wofunsidwayo wayankha kuti zimveke bwino ndipo funsisitsani mozama pa mutu uli onse. Machezawa adziyenda mooneka ngati mukungocheza osati ngati mukumufunsa chinthu munthu. - Kuchokera apa, machezawa atengedwe ndi chintapa mawu koma mukhale mutatenga chilolezo kuchokera kwa yemwe mukucheza nayeyo. - Lembani zomwe mukuona m’munsi mwa pepala lomweli patsiku lamacheza lomwelo. |
| 1. *[Mafunso oyambilira:]* Poyamba pa kucheza kwathu lero, mungandiuze dzina lanu? Mungandifotokozere za banja lanu?   *[Funsisitsani]: Mnyumba mwanu mumakhala ndani? Mwakhala mdera lino kwanthawi yaitali bwanji? Mumagwira ntchito yanji? Mumakonda kupanga chiyani mukakhala ndi danga?*  *Monga momwe ndinanenera poyamba paja [onani pepala la chilolezo], tsopano ndikufunsani mafunso okhudzana ndi kachilombo ka HIV. Ndikhulupilira kuti izi ndizabwinobwino kwainu ndipo kuti mukhala omasuka ndi machezawa. Pongofuna kukukumbutsani, ngati mungamve kumangika chifukwa cha funso lina liri lonse, simuli okakamizidwa kuliyankha ndipo tidzapita pa funso lina.*   1. Mwakhala mukulandira chithandizo cha kachilombo ka HIV pachipatala chino kapena malo ano kwa nthawi yayitali bwanji?   *[Funsisitsani]: Kodi kano ndikoyamba kubwera kuchithandizo cha kachilombo ka HIV?*  *Munayamba mwapitako kuchipatala china m’mbuyomu? Ngati munapitapo, chipatala chake ndichiti ndipo munapita kwa nthawi yayitali bwanji?*  *Munasankhilanji kubwera pachipatala chino?*   1. Mungalongosole chithandizo chomwe mukulandira pakali pano chokhudzana ndi kachilombo ka HIV?   *[Funsitsani]: Kodi mukulandira chithandizo china choonjezera kuchokera ku banja lanu, okondedwa anu, ogwira ntchito ya zaumoyo, alangizi, anzanu ndi ena otero?*   1. Tiyeni tibwelerenso kunthawi yomwe munapezeka ndi kachilombo ka HIV, ngati simukuona vuto kutero. Mungandiuzeko zambiri za nthawi imene munapezeka ndi kachilombo?   *[Funsisitsani]: Chifukwa chiyani munakayezetsa? Munakakmizika kutero? Munachita kusankha kuti muyezetse? Munakayezetsa ndi munthu wina? Munakayezetsa ndi okondedwa anu?*  *Munatani mutamva zotsatira?*  *Munamuuza ndani zotsatira?*  *Munali ndi chithandizo chotani pa nthawi imeneyo, ngati chinalipo?*   1. Munapemphedwa kuti muyambe kumwa mankhwala otalikitsa moyo a ma ARV?   *[Funsisitsani]: Ndiliti lomwe munauzidwa kuti muyambe kumwa mankhwala otalikitsa moyo a ma ARV?*  *Anakufotokozerani chiyani nthawi imeneyo?*  *Mukuyenera kumwa mankhwalawa kwanthawi yayitali bwanji?*  *Munamva bwanji zoyamba kumwa mankhwala panthawi imeme munayamba?*  *Mukanasankha kuyamba kumwa mankhwala? [Funsisitsani]: Munapanga chiganizo chotani panthawi imeneyo? Chinakupangitsani ndi chiyani?*   1. Mungafotokoze zomwe zinachitika mutapemphedwa kuti muyambe kumwa mankhwala a kachilombo ka HIV?   *[Funsisitsani]: Kodi mwakambilanapo ndindani za mankhwala a kachilombo ka HIV?*  *Panapita nthawi yayitali bwanji mutayezetsa kuti muyambe kumwa mankhwala kwanthawi yoyamba?*  *Munapitilizabe kumwa mankhwala? Ngati zimunapitilize, mungafotokoze kuti chinachitika ndi chiyani? [Funsisitsani za zifukwa zosapitilizira, ndipomwe anasiyira kumwa mankhwala, kuyambanso kapena kusiyitsidwa]?*   1. Kodi mumamwa makhwala pafupipafupi?   *[Funsisitsani]: Mumamva/mumaona bwanji za mankhwala a HIV?*  *Ngati nthawi zina simumamwa mankhwala, zifukwa zake ndichiyani?*   1. Kodi munakambirana ndi okondedwa anu zoyezetsa ngati muli ndi kachilombo ka HIV?   *[Funsisitsani]: Nanga za mankhwala anu a kachilombo ka HIV, munakambirana ndi okondedwa anu?*  *Kodi okondedwa anu anayezetsapo ngati alii ndi kachilombo ka HIV? Ngati anayezetsapo, anakuuzani za zotsatira za kuyezetsako?*  *Kodi mukudziwa ngati okondedwa anu akumwa mankhwala a kachilombo ka HIV panopa?*  *Kodi munayamba mwagawanapo mankhwala anu a kachilombo ka HIV ndi okondedwa anu kapena munthu wina? [Ngati anayamba agawanapo, funsisitsani: chifukwa chiyani, ndi ndani, afotokoze momwe izi zinayendera, kodi kanali kamodzi kokha kapena zimapangika kawirikawiri?]*  *Ndi ndani wina yemwe munamudziwitsa kapena kukambirana naye za momwe mthupi mwanu mulili? [Funsisitsani: ndili lomwe munawauza anthuwa? Munatsata ndondomeko yotani? Izi zinachitikira kuti? Anatani atamva izi?]*   1. Munalandila malangizo otani okhudzana ndi kuyamwitsa?   *[Funsisitsani]: Kuyambira pomwe munayamba kumwa mankhwala otalikitsa moyo, chilipo china chili chonse chokhudzana ndi kuyamwitsa chomwe mwasinthapo?*  *[Funsisitsani kuti mudziwe ngati maganizo kapena mayamwitsidwe asintha chiyambire kumwa mankhwala otalikitsa moyo]*   1. Kodi mudzafuna kukhalanso ndi pakati mtsogolo muno?   *[Funsisitsani]: Kodi kumwa mankhwala otalikitsa moyo kunakupangitsani kukhala ndimalingaliro ena pa zodzakhala ndi pakati mtsogolo muno?*  *[Funsisitsani kuti mumvetse ngati chithandizo mankhwala otalikitsa moyo chapangitsa kuganiza kuti ndizoopsya kapena zosaopsya kukhala ndi pakati pena]*   1. M’maganizo mwanu, pali zinthu zomwe ziyenera kusinthidwa kuti ntchito yoyeza magazi ngati muli kachilombo ndiyoyambitsa mankhwala a kachilombo ka HIV kuti ipite patsogolo?   *[Funsisitsani]: Kodi munali omasuka kuyamba kumwa mankhwala akachilombo ka HIV mutangoyezetsa?*  *M’maganizo mwanu, ndichithandizo chotani chomwe chiyenera kuperekedwa kwa amayi ndi mabanja awo ngati mbali imodzi yachithandizo cha kachilombo ka HIV?* |
| **Ndemanga zowonjezera**  *Tisanamalize kucheza muli ndi ndemanga ina iliyonse yokhudzana ndi nkhani yomwe takambilanayi? [Fotokozani]* |
| **Malangizo kwa ofunsa mafunso:**   - Athokozeni omwe mumacheza naowo chifukwa cha nthawi yawo ndipo akumbutseni kuti tidzafuna kucheza nawonso pakadutsa miyezi isanu ndi umodzi ngati angavomere. - Mukatha machezawo (tsiku lomwelo) lembani zomwe mumaona nthawi yamachezawo kuti zithandizile kufotokozela bwino zomwe mwapeza (mwachitsanzo malo, momwe machezawo amayendera, ngati panali kudulidwa, kukhudzidwa, kudodoma, maonekedwe a ofunsidwawo ndi zina zotero.) |

### Interview Guide for LTFU women (Chichewa)

| **Dongosolo lofunsira mafunso: AMAYI OMWE SAKUPEZEKA**  Kafukufuku wa NEMAPP |
| --- |
| **Nambala ya**  **macheza: Boma:**  **Chipatala/Malo:**  **Tsiku lolowera mukafukufuku: (Tsiku) (Mwezi)**  **Nambala yamu kafukufuku**  **wa NEMAPP:**  **Zaka za ofunsidwa mafunso:**  **Ndiokwatiwa kapena**  **osakwatiwa:**  1= Okwatiwa  2= Nkazi wa mamasiye  3= Anasiyana  4= Sanakwatiwepo  5= China (longosoloni)________________________________  **Maphunziro**  1= Sanaphunzire  2= Makalasi am’munsi ku pulaimale  3= Makalasi a m’mwamba ku pulaimale  4= Makalasi a m’munsi ku sekondale  5= Makalasi a m’mwamba kusekondale  6= Adafika pa ukachenjede  7= Satifiketi kapena Dipuloma (longosolani)__________________________  8= China (longosolani)____________________________________________________  **Mtundu**  1 = Muyao  2= Mulomwe  3= Mchewa  4= Mtumbuka  5= Mngoni  6= Msena  7= Wina (longosolani)________________________________  **Chipembedzo:**  1= Chikhilisitu  2= Chisilamu  3= Chikhalidwe  4= Palibe kapena china (longosolani) ___________________________  **Ali ndi ana angati:**  **Ndiwoyembekezera?**  1= Inde  2= Ayi  **Akuyamwitsa? If yes Ngati akuyamwitsa, anayamba liti?**  1=Inde  2= Ayi  **Ofunsa mafunso/Otsogolera:**    **HRS**  **HRS**  **Tsiku: Nthawi yoyambira: Nthawi yomalizira:** |
| **Malangizo kwa ofunsa mafunso:**   - Tsalirani malamulo otengela chilolezo kuphatikizapo chilolezo chotenga macheza ndi chintapa mau. Mupatseni yemwe mukumufunsa mafunsoyo nthawi yokwanira yoti apangire chiganizo. Yemwe mukucheza nayeyo akhale omasuka kucheza nanu nthawi yopitilira ola limodzi (Ngati sangathe, pezani tsiku lina). - Mukuyenera kuwonetsetsa kuti chinsinsi chisungika (kuphatikizapo chinsinsi kuti zomwe zikulankhulidwa zisamveke) pa nthawi yomwe mukucheza. - Ili ndi dongosolo logwiritsidwa ntchito pa zokambirana zozama (osati pongofufuza pang’ono): mukuyenera kufunsa momwe mungathere pa zomwe wofunsidwayo wayankha kuti zimveke bwino ndipo funsisitsani mozama pa mutu uli onse. Machezawa adziyenda mooneka ngati mukungocheza osati ngati mukumufunsa chinthu munthu. - Kuchokera apa, machezawa atengedwe ndi chintapa mawu koma mukhale mutatenga chilolezo kuchokera kwa yemwe mukucheza nayeyo. - Lembani zomwe mukuona m’munsi mwa pepala lomweli patsiku lamacheza lomwelo. |
| *1. [Mafunso oyambilira:]* Poyamba pa kucheza kwathu lero, mungandiuze dzina lanu? Mungandifotokozere za banja lanu?  *[Funsisitsani]: Mnyumba mwanu mumakhala ndani? Mwakhala mdera lino kwanthawi yaitali bwanji? Mumagwira ntchito yanji? Mumakonda kupanga chiyani mukakhala ndi danga?*  *Monga momwe ndinanenera poyamba paja [onani pepala la chilolezo], tsopano ndikufunsani mafunso okhudzana ndi kachilombo ka HIV. Ndikhulupilira kuti izi ndizabwinobwino kwainu ndipo kuti mukhala omasuka ndi machezawa. Pongofuna kukukumbutsani, ngati mungamve kumangika chifukwa cha funso lina liri lonse, simuli okakamizidwa kuliyankha ndipo tidzapita pa funso lina.*   1. Taona kuti simukupitiliza kudzalandila chithandizo chokhudzana ndi kachilombo ka HIV. Zifukwa zomwe munaganizira kuti musapitilize kulandira chithandizochi ndi chiyani?   *[Funsisitsani]: Mungafotokoze za momwe munkamvera lapena kuonera za chithandizo cha kachilombo ka HIV ku chipatala chomwe munkapita?*  *Mungafotokoze za momwe munaverera kapena kuonera za chithandizo cha mankhwala a kachilombo ka HIV?*   1. Munalandila chithandizo cha kachilombo ka HIV pachipatalachi kwanthawi yayitali bwanji musanasiye?   *[Funsisitsani]: Kodi iyi inali nthawi yoyamba kuti mulandile chithandizo cha kachilombo ka HIV?*  *Munapitako kuchipatala china m’mbuyomu? Ngati ndichoncho, chipatala chake chinali chiti ndipo munapita kwanthawi yayitali bwanji?*  *Chifukwa chiyani munasankha kubwera kuchipatala chino?*   1. Mungalongosole chithandizo chomwe mukulandira pakali pano chokhudzana ndi kachilombo ka HIV?   *[Funsitsani]: Kodi mukulandira chithandizo china choonjezera kuchokera ku banja lanu, okondedwa anu, ogwira ntchito ya zaumoyo, alangizi, anzanu ndi ena otero?*   1. Tiyeni tibwelerenso kunthawi yomwe munapezeka ndi kachilombo ka HIV, ngati simukuona vuto kutero. Mungandiuzeko zambiri za nthawi imene munapezeka ndi kachilombo?   *[Funsisitsani]: Chifukwa chiyani munakayezetsa? Munakakmizika kutero? Munachita kusankha kuti muyezetse? Munakayezetsa ndi munthu wina? Munakayezetsa ndi okondedwa anu?*  *Munatani mutamva zotsatira?*  *Munamuuza ndani zotsatira?*  *Munali ndi chithandizo chotani pa nthawi imeneyo, ngati chinalipo?*   1. Munapemphedwa kuti muyambe kumwa mankhwala otalikitsa moyo a ma ARV?   *[Funsisitsani]: Ndiliti lomwe munauzidwa kuti muyambe kumwa mankhwala otalikitsa moyo a ma ARV?*  *Anakufotokozerani chiyani nthawi imeneyo?*  *Mukuyenera kumwa mankhwalawa kwanthawi yayitali bwanji?*  *Munamva bwanji zoyamba kumwa mankhwala panthawi imeme munayamba?*  *Mukanasankha kuyamba kumwa mankhwala? [Funsisitsani]: Munapanga chiganizo chotani panthawi imeneyo? Chinakupangitsani ndi chiyani?*   1. Mungafotokoze zomwe zinachitika mutapemphedwa kuti muyambe kumwa mankhwala a kachilombo ka HIV?   *[Funsisitsani]: Kodi mwakambilanapo ndindani za mankhwala a kachilombo ka HIV?*  *Panapita nthawi yayitali bwanji mutayezetsa kuti muyambe kumwa mankhwala kwanthawi yoyamba?*  *Munapitilizabe kumwa mankhwala? Ngati zimunapitilize, mungafotokoze kuti chinachitika ndi chiyani? [Funsisitsani za zifukwa zosapitilizira, ndipomwe anasiyira kumwa mankhwala, kuyambanso kapena kusiyitsidwa]?*   1. Taona kuti simunapitenso kuchipatala pakatipa. Kodi mumapitiliza kumwa mankhwala koma osapitanso kuchipatala kapena munasiya?   *[Funsisitsani]: Ngati mumamwabe mankhwala, mumawatenga kuti?*  *[Kodi munaona kapena mumaona bwanji za chithandizo cha mankhwala a kachilombo ka HIV?*  *Ngati simukumwa mankhwala pakali pano, zifukwa zake ndi zotani?*   1. Kodi munakambirana ndi okondedwa anu zoyezetsa ngati muli ndi kachilombo ka HIV?   *[Funsisitsani]: Nanga za mankhwala anu a kachilombo ka HIV, munakambirana ndi okondedwa anu?*  *Kodi okondedwa anu anayezetsapo ngati alii ndi kachilombo ka HIV? Ngati anayezetsapo, anakuuzani za zotsatira za kuyezetsako?*  *Kodi mukudziwa ngati okondedwa anu akumwa mankhwala a kachilombo ka HIV panopa?*  *Kodi munayamba mwagawanapo mankhwala anu a kachilombo ka HIV ndi okondedwa anu kapena munthu wina? [Ngati anayamba agawanapo, funsisitsani: chifukwa chiyani, ndi ndani, afotokoze momwe izi zinayendera, kodi kanali kamodzi kokha kapena zimapangika kawirikawiri?]*  *Ndi ndani wina yemwe munamudziwitsa kapena kukambirana naye za momwe mthupi mwanu mulili? [Funsisitsani: ndili lomwe munawauza anthuwa? Munatsata ndondomeko yotani? Izi zinachitikira kuti? Anatani atamva izi?]*   1. Munalandila malangizo otani okhudzana ndi kuyamwitsa?   *[Funsisitsani]: Kuyambira pomwe munayamba kumwa mankhwala otalikitsa moyo, chilipo china chili chonse chokhudzana ndi kuyamwitsa chomwe mwasinthapo?*  *[Funsisitsani kuti mudziwe ngati maganizo kapena mayamwitsidwe asintha chiyambire kumwa mankhwala otalikitsa moyo]*   1. Kodi mudzafuna kukhalanso ndi pakati mtsogolo muno?   *[Funsisitsani]: Kodi kumwa mankhwala otalikitsa moyo kunakupangitsani kukhala ndimalingaliro ena pa zodzakhala ndi pakati mtsogolo muno?*  *[Funsisitsani kuti mumvetse ngati chithandizo mankhwala otalikitsa moyo chapangitsa kuganiza kuti ndizoopsya kapena zosaopsya kukhala ndi pakati pena]*   1. M’maganizo mwanu, pali zinthu zomwe ziyenera kusinthidwa kuti ntchito yoyeza magazi ngati muli kachilombo ndiyoyambitsa mankhwala a kachilombo ka HIV kuti ipite patsogolo?   *[Funsisitsani]: Kodi munali omasuka kuyamba kumwa mankhwala akachilombo ka HIV mutangoyezetsa?*  *M’maganizo mwanu, ndichithandizo chotani chomwe chiyenera kuperekedwa kwa amayi ndi mabanja awo ngati mbali imodzi yachithandizo cha kachilombo ka HIV?* |
| **Ndemanga zowonjezera**  *Tisanamalize kucheza muli ndi ndemanga ina iliyonse yokhudzana ndi nkhani yomwe takambilanayi? [fotokozani]* |
| **Malangizo kwa ofunsa mafunso:**   - Athokozeni omwe mumacheza naowo chifukwa cha nthawi yawo ndipo akumbutseni kuti tidzafuna kucheza nawonso pakadutsa miyezi isanu ndi umodzi ngati angavomere. - Mukatha machezawo (tsiku lomwelo) lembani zomwe mumaona nthawi yamachezawo kuti zithandizile kufotokozela bwino zomwe mwapeza (mwachitsanzo malo, momwe machezawo amayendera, ngati panali kudulidwa, kukhudzidwa, kudodoma, maonekedwe a ofunsidwawo ndi zina zotero.) |

### Focus group discussion guide for Health care workers (Chichewa)

| **Malangizo a macheza a pagulu: Ogwira ntchito ya zaumoyo (omwe ali mu ndondomeko ya Option B+)**  Kafukufuku wa NEMAPP | | | | |
| --- | --- | --- | --- | --- |
| **Nambala ya macheza a pagulu: Boma:**  **Chipatala:**  **AM/PM**  **AM/PM**  **Tsiku: Nthawi yoyambira: Nthawi yomalizira:** | | | | |
|  | **Zaka** | **Mamuna kapena Mkazi** | **Maphunziro** | **Udindo (Namwino, Mlangizi, Dotolo, ndi zina zotero.)** |
| 1 |  |  |  |  |
| 2 |  |  |  |  |
| 3 |  |  |  |  |
| 4 |  |  |  |  |
| 5 |  |  |  |  |
| 6 |  |  |  |  |
| 7 |  |  |  |  |
| 8 |  |  |  |  |
| 9 |  |  |  |  |
| 10 |  |  |  |  |
| **Maphunziro**  1= Sanaphunzire 2= Makalasi am’munsi ku pulaimale 3= Makalasi a m’mwamba ku pulaimale 4= Makalasi a m’munsi ku sekondale  5= Makalasi a m’mwamba kusekondale 6= Adafika pa ukachenjede 7= Satifiketi kapena Dipuloma (longosolani)__________________________  8= China (longosolani)____________________________________________________  **Opangitsa macheza:** | | | | |
| **Malangizo kwa otsogolera macheza apagulu:**   - Malangizowa akuthandizani kuyambitsa ndikutsogolera machezawa, omwe odzaoneka ngati kukambirana kwanthawi zonse osati ngati kufunsana mafunso. - Malangizowa alembedwa kuti atsogolere gulu la anthu pakati pa anayi ndi asanu ndi m’modzi omwe ayenera kupangika kamodzi pachipatala chili chonse. Ndichabwino kuti pagulu liri lonse padzikhala amuna ndi akazi. - Opanga nawo machezawa akuyenera kudziwa kuti kukambiranaku kutenga pafupifupi mphindi makumi sanu ndi limodzi (60). (Ngati zizingatheke kutero, pezani tsiku lina). - Funsani m’modzi mwaopangitsa kafukufuku kuti adzilemba za momwe gululi likuchitira, momwe gululi likugwirizanila ndi zomwe gululi likukambirana. - Onetsetsani kuti chinsinsi chisungika (kuphatikizapo chinsinsi kuti zomwe zikulankhulidwa zisamveke) pa nthawi yomwe mukucheza. Nkutheka kuti anthu amguli akudziwana kale. Khazikitsani malamulo kuti zonse zomwe mukambirane pagulupo zikhale zachinsinsi. | | | | |
| Tikufuna tikambirane za momwe ntchito yoteteza mwana kuti asatengere kacholombo kuchokera kwa mayi imakonzedwera ndi kumvetsera zomwe inu mumaona tsiku ndi tsiku pa zochitika pachipatala pano.   1. Maganizo anu ndi otani pachithandizo chomwe chikuperekedwa pachipatala pano, zikuyenda bwino ndi ziti ndipo zimene zikufunika zitasintha ndi ziti? 2. Mungafotokoze motani zimene azimayi amakumana nazo akamalandila chithandizo choperekedwa kwa azimayi kuti mwana obadwayo asatengere kachilombo (PMTCT) pachipatala pano? 3. Mumaganizo anu mukuwona kuti tingapange chani kuti azibambo azitha kutenga nawo mbali polandira chithandizo choperekedwa kwa mayi kuti asapereke kachilombo kwa mwana? 4. Pali chithandizo china chimene inuyo mukuwona kuti chikusoweka? Pali chithandizo chilichonse chimene mukuwona kuti ndichofunika pachipatala pano? 5. Mumayiwona bwanji ndondomeko yoyambitsira mankhwala otalikitsa moyo?   M’maganizo anu, ndichani chomwe chikuyenera kusinthidwa mundondomeko yoyambitsira mankhwala?     1. Mungafotokoze momwe mumaonela ndondomeko yopereka mankhwala otalikitsa moyo kwa mayi wina aliyense oyembekezera kapena oyamwitsa yemwe ali ndi kachilombo?   Kodi pali chomwe ndondomekoyi imasintha pa zokhudzana ndi chithandizo chamankhwala a kachilombo ka HIV, kuyezetsa, kuyamwitsa ndikuti azibambo azitha kutenga nawo mbali?   1. M’maganizo anu, mukuganiza kuti ndichifukwa chiyani amayi ena samabwelanso?   Ndizifukwa zznji zomwe ena sabweleranso? Kodi ndondomeko yoteteza mwana kuti asatengere kachilombo kwa mayi ingathandize bwanji kuchepetsa chiwerengero cha amayi omwe samabweleranso kuchipatala?   1. Mmaganizo anu mukuwona ngati ndichithandizo chanji chapamwamba chimene chingaperekedwe kwa amayi woyamwitsa komanso amayi woyembekezera chokhuzana ndi ndondomeko ya PMTCT? Kodi ndi ntchito yanji yazaumoyo yofunikira kwambiri yomwe chipatala chino chikugwira pakadali pano? (Fotokozani) | | | | |
| **Malangizo kwa otsogolera macheza apagulu:**   - Athokozeni omwe mumacheza naowo chifukwa cha nthawi yawo ndipo akumbutseni kuti tidzafuna kucheza nawonso pakadutsa miyezi isanu ndi umodzi ngati angavomere. - Ttsiku lomwelo, lembani zomwe mumaona nthawi yamachezawo zokambilanazo zikangotha – lembani za momwe gululo limakhalira nthawi ya zokambiranazo (mongamalo, kupikisana mphamvu, kudukizadukiza ndi zina zili zonse zofunika kuti zithandizile kufotokozela bwino zomwe mwapeza.) | | | | |

### Interview Guide for Male Partners (Chichewa)

| **Ndondomeko yofunsira mafunso: Okondedwa a amayi omwe alowa mukafukufuku**  **Kafukufuku wa NEMAPP** |
| --- |
| **Nambala ya**  **macheza: Boma:**  **Chipatala/Malo:**  **Tsiku lolowera mukafukufuku: (Tsiku) (Mwezi)**  **Nambala yamu kafukufuku**  **wa NEMAPP:**  **Zaka za ofunsidwa mafunso:**  **Ndiokwatiwa kapena**  **osakwatiwa:**  1= Okwatiwa  2= Nkazi wa mamasiye  3=Anasiyana  4=Sanakwatiwepo  5=China (longosoloni)________________________________  **Maphunziro**  1= Sanaphunzire  2=Makalasi am’munsi ku pulaimale  3= Makalasi a m’mwamba ku pulaimale  4=Makalasi a m’munsi ku sekondale  5=Makalasi a m’mwamba kusekondale  6=Adafika pa ukachenjede  7=Satifiketi kapena Dipuloma (longosolani)__________________________  8=China (longosolani)____________________________________________________  **Mtundu**  1= Muyao  2= Mulomwe  3= Mchewa  4= Mtumbuka  5= Mngoni  6= Msena  7= Wina (longosolani)________________________________  **Chipembedzo:**  1= Chikhilisitu  2= Chisilamu  3= Chikhalidwe  4 = Palibe kapena china (longosolani) ___________________________  **Ali ndi ana angati:**  **Mayi akunyumba**  **ndiwoyembekezera?**  1= Inde  2= Ayi  **Akuyamwitsa? If yes Ngati akuyamwitsa, anyamba liti?**  1=Inde  2= Ayi  **Ofunsa mafunso/Otsogolera:**    **HRS**  **HRS**  Tsiku Nthawi yoyambira Nthawi yomalizira |
| **Malangizo kwa ofunsa mafunso:**   - Tsalirani malamulo otengela chilolezo kuphatikizapo chilolezo chotenga macheza ndi chintapa mau. Mupatseni yemwe mukumufunsa mafunsoyo nthawi yokwanira yoti apangire chiganizo. Yemwe mukucheza nayeyo akhale omasuka kucheza nanu nthawi yopitilira ola limodzi (Ngati sangathe, pezani tsiku lina). - Mukuyenera kuwonetsetsa kuti chinsinsi chisungika (kuphatikizapo chinsinsi kuti zomwe zikulankhulidwa zisamveke) pa nthawi yomwe mukucheza. - Ili ndi dongosolo logwiritsidwa ntchito pa zokambirana zozama (osati pongofufuza pang’ono): mukuyenera kufunsa momwe mungathere pa zomwe wofunsidwayo wayankha kuti zimveke bwino ndipo funsisitsani mozama pa mutu uli onse. Machezawa adziyenda mooneka ngati mukungocheza osati ngati mukumufunsa chinthu munthu. - Kuchokera apa, machezawa atengedwe ndi chintapa mawu koma mukhale mutatenga chilolezo kuchokera kwa yemwe mukucheza nayeyo. - Lembani zomwe mukuona m’munsi mwa pepala lomweli patsiku lamacheza lomwelo. |
| 1. Panopa mukumalandira chithandizo chilichonse kuchipatala chimene okondedwa anu amalandirako chisamaliro? (Funsisitsani kuti ndichithandizo chiti) 2. Ndifotokozereni ngati munayamba mwayezetsapo magazi anu kuti mudziwe ngati muli ndikachirombo ka HIV komanso fotokozani ngati munalandirapo chithandizo china chili chonse musanayezetse kapena mutayezetsa magazi? 3. Mungafotokozepo za chithandizo chomwe okondedwa anu akulandira panopa kumbali ya HIV? [Funsisitsani: ndichithandizo chanji chimene akulandira kuchokera kwa inuyo ngati wokondedwa awo, kuchokera kwa ogwira ntchito pachipatala, kuchokera kwa banja lawo, kuchokera kwa alangizi ndiponso kuchokera kwa anzawo wocheza nawo?) 4. Kodi chithandizo chimene okondedwa anu amalandira kumbali ya HIV, kuphatikizapo mankhwala otalikitsa moyo ndi chosiyana ndi zithandizo zina zimene zikuperekedwa pachipatala chino? [Ngati zikusiyana zikusiyana bwanji?]   5. Mukuwona ngati chithandizo chomwe okondedwa anu amalandira chingakhudze moyo wanu? (Funsisitsani: chingakhudze bwanji moyo wanu)  6. Kodi inuyo panopa mukulandira chithandizo chofanana ndi chimene okondedwa anu akulandira? (Funsisitsani) |
| ***7.*** [*Ngati anayezetsapo*]: Mungandifotokozere za nthawi imene munayezetsa magazi? [Funsisitsani: Chifukwa chani mudakayezetsa? Munazilandila bwanji zotsatira za kuyezetsa magazi anu? Munamufotokozera ndani za zotsatira zanu ndipo monalandira chithandizo chanji nthawi imeneyo?]  ***8.*** [*Ngati anayezetsapo ndikupezeka ndikachilombo*]: Ndi liti lomwe munawuzidwa kuti muyambe kulandila mankhwala otalikitsira moyo (ma ARV)? [Funsisitsani: *Zinatenga nthawi yayitali bwanji kuchokera nthawi yomwe munayezetsa kuti muyambe kumwa mankhwala wotalikitsa moyowa (ma ARVs)*]   1. [*Ngati akulandila mankhwala*]: Tandifotokozereni za zomwe mwakhala mukukumana nazo kuyambira pomwe munayezetsa magazi ndi kuyamba kulandira mankhwala otalikitsa moyo mpaka pano 2. [Ngati akumwa mankhwala]: Mukulandila/mukumwa mankhwala mu nthawi mwake? [*Funsisitsani: Ndizovuta ziti zimene mumakumana nazo mukamamwa mankhwala amene amaperekedwa kwa anthu amene ali ndi kachirombo ka HIV?*] 3. Kodi mukudziwa za zovuta zimene okondedwa anu amakumana nazo pamene akulandira/kumwa mankhwala amene amaperekedwa kwa anthu amene ali ndi kachirombo? [Funsisitsani].   Kodi mulindi maganizo ofuna kuzaberekaso mtsogolomu? [Funsisitsani: Mungafotokoze zifukwa zomwe zikukupangitsani kuti mufune kapena musafune kukhala ndi ana ena mutsogolomu? Mukuwona ngati kulandira mankhwala otalikitsa moyo kwa okondedwa anu kukukhudzana bwanji ndi nkhani yokhala ndi ana ochuluka mtsogolo?]   1. Mmaganizo anu ndizinthu ziti zomwe ziyenera kusintha kuti ntchito yoyezetsa magazi ndi kuyamba kumwa mankhwala otalikitsa moyo ipite patsogolo? 2. Munakumanako ndi azibambo omwe amakana kuyezetsa magazi kapena kuyamba kumwa mankhwala otalikitsa moyo (ARV)? [Funsistsani: Ndichifukwa chiyani amakana kuyamba kumwa mankhwala?]   15. Mumaganizo anu ndi chithandizo chanji chomwe chiyenera kuperekedwa kwa amayi omwe ali ndi kachilombo ka HIV komanso mabanja awo ngati mbali ya chithandizo choperekedwa kwa anthu amene ali ndi kachilombo ka HIV? |
|  |
| 1. [Ngati anayezetsapo]: Ndi anthu ati amene munawafotokozera za mmene mulili mthupi mwanu?(munawafotokozera liti anthu amenewa)   ***17.*** [Ngati anayezetsapo]: Tatifotokozereni ndondomeko yomwe munatsatira powawulurila anthu amenewa za mmene mthupi mwanu mulili? Munawafotokozera bwanji, kuti (malo) ndipo anautenga bwanji uthengawu mutawafotokozera?  ***18.*** [Ngati anayezetsapo]: Kodi zotsatira za kuyetsa magazi kwanu munawafotokozera okondedwa anu (ngati simunawafotokozere nchifukwa chiyani simunatero, ngati munawafotokozera munatsata ndondomeko yanji powafotokozera?   1. Mumaganizo anu mukuwona ngati ndi chinthu chofunikira kuti munthu awulure kwa anthu ena za mmene mthupi mwanu mulili? Ndi chifukwa chani mukuwona kuti ndi zofunikira? [Funsisitsani pa zovuta zomwe angatchule] |
| Tisanamalize kucheza pali ndemanga ina iliyonse imene mulinayo yokhudzana ndi zomwe takambiranazi? |
| **Malangizo kwa ofunsa mafunso:**   - Athokozeni omwe mumacheza naowo chifukwa cha nthawi yawo ndipo akumbutseni kuti tidzafuna kucheza nawonso pakadutsa miyezi isanu ndi umodzi ngati angavomere. - Mukatha machezawo (tsiku lomwelo) lembani zomwe mumaona nthawi yamachezawo kuti zithandizile kufotokozela bwino zomwe mwapeza (mwachitsanzo malo, momwe machezawo amayendera, ngati panali kudulidwa, kukhudzidwa, kudodoma, maonekedwe a ofunsidwawo ndi zina zotero.) |

### Interview Guide for Community Leaders (Chichewa)

| **Dongosolo lofunsira mafunso: Atsogoleri**  Kafukufuku wa NEMAPP |
| --- |
| **Nambala ya**  **macheza: Boma:**  **Chipatala/Malo:**  **Tsiku lolowera mukafukufuku: (Tsiku) (Mwezi)**  **Nambala yamu kafukufuku**  **wa NEMAPP:**  **Zaka za ofunsidwa mafunso:**  **Ndiokwatiwa kapena**  **osakwatiwa:**  1= Okwatiwa  2= Nkazi wa mamasiye  3= Anasiyana  4= Sanakwatiwepo  5= China (longosoloni)________________________________  **Maphunziro**  1= Sanaphunzire  2= Makalasi am’munsi ku pulaimale  3= Makalasi a m’mwamba ku pulaimale  4= Makalasi a m’munsi ku sekondale  5= Makalasi a m’mwamba kusekondale  6= Adafika pa ukachenjede  7= Satifiketi kapena Dipuloma (longosolani)__________________________  8= China (longosolani)____________________________________________________  **Mtundu**  1= Muyao  2= Mulomwe  3= Mchewa  4= Mtumbuka  5= Mngoni  6= Msena  7= Wina (longosolani)________________________________  **Chipembedzo:**  1= Chikhilisitu  2= Chisilamu  3= Chikhalidwe  4 = Palibe kapena china (longosolani) ___________________________  **Mamuna/Nkazi:**  **Ofunsa mafunso/Otsogolera:**  **TSIKU/MWEZI/CHAKA**  **HRS**  **HRS**  **Tsiku: Nthawi yoyambira: Nthawi yomalizira:** |
| **Malangizo kwa ofunsa mafunso:**   - Tsalirani malamulo otengela chilolezo kuphatikizapo chilolezo chotenga macheza ndi chintapa mau. Mupatseni yemwe mukumufunsa mafunsoyo nthawi yokwanira yoti apangire chiganizo. Yemwe mukucheza nayeyo akhale omasuka kucheza nanu nthawi yopitilira ola limodzi (Ngati sangathe, pezani tsiku lina). - Mukuyenera kuwonetsetsa kuti chinsinsi chisungika (kuphatikizapo chinsinsi kuti zomwe zikulankhulidwa zisamveke) pa nthawi yomwe mukucheza. - Ili ndi dongosolo logwiritsidwa ntchito pa zokambirana zozama (osati pongofufuza pang’ono): mukuyenera kufunsa momwe mungathere pa zomwe wofunsidwayo wayankha kuti zimveke bwino ndipo funsisitsani mozama pa mutu uli onse. Machezawa adziyenda mooneka ngati mukungocheza osati ngati mukumufunsa chinthu munthu. - Kuchokera apa, machezawa atengedwe ndi chintapa mawu koma mukhale mutatenga chilolezo kuchokera kwa yemwe mukucheza nayeyo. - Lembani zomwe mukuona m’munsi mwa pepala lomweli patsiku lamacheza lomwelo. |
| 1. Maganizo anu ndi otani pachithandizo chomwe chikuperekedwa pachipatala cha m’dera lino?   *[Funsisitsani]*: Zikuyenda bwino ndi ziti? Zikufunika zitasintha ndi ziti?   1. Mungafotokoze motani zimene azimayi amakumana nazo akamalandila chithandizo choperekedwa kwa azimayi kuti mwana obadwayo asatengere kachilombo (PMTCT) pachipatala chomwe anthu am’mudzi mwanu amapita?     [*Funsisitsani*]: Kodi azimayi amakudziwani, ndipo amayi omwe amalandira chithandizo kudzera mu PMTCT anayamba akuuzanipo maganizo kapena awo nkhawa zawo?   1. Mumaganizo anu mukuwona kuti chikupangika ndi chani kapena chongamapangike ndi chani kuti azibambo adzitha kutenga nawo mbali polandira chithandizo choperekedwa kwa mayi kuti asapereke kachilombo kwa mwana? 2. Pali chithandizo china chimene inuyo mukuwona kuti chikusoweka? Pali chithandizo chilichonse chimene mukuwona kuti ndichofunika kwa amayi ndi mabanja awo? 3. Mukuyiwona bwanji ndondomeko yoyambitsira mankhwala otalikitsa moyo kwa odwala omwe ali ndi kachilombo ka HIV?   *[Funsisitsani]:* M’maganizo anu, ndichani chomwe chikuyenera kusinthidwa mundondomeko yoyambitsira mankhwala?   1. Kodi mawu okuti ‘Option B+’ mumawadziwa? Ngati mumawadziwa, mungafotokoze momwe mmaganizira kuti amatanthauza chiyani?   *[Funsisitsani]:* Kuchokera momwe inu mukuonera, kodi ndondomekoyi imakhudza bwanji zokhudzana ndi chithandizo cha mankhwala a kachilombo ka HIV, kuyezetsa, kuyamwitsa ndi kuti abambo adzitengapo mbali?   1. Kodi mukuganiza kuti kulandila mankhwala otalikitsa moyo a ma ARV kungakhudze kuyamwitsa m’dera lino?   *[Funsisitsani]:* Zingakhudze bwanji kuyamwitsa ndipo zingakhudze kwambiri ndani?   1. Kodi mukuganiza kuti kulandila mankhwala otalikitsa moyo a ma ARV kungakhudze chifuniro cha mzimayi chofuna kudzakhalanso ndipakati m’dera lino?   *[Funsisitsani]:* Zingakhudze motani kukhala ndi pakati mtosgolo ndipo zingakhudze ndani kwambiri?   1. M’maganizo mwanu, mukuganiza kuti ndi chifukwa chiyani azimayi ena samabweranso kuchipatala kudzatenga mankhwala kapena samabweranso pa tsiku lomwe anawauza kuti abwerenso mzipatala za m’dera lino?   *[Funsisitsani]:* M’mene mumawonera inu, ndizifukwa ziti zomwe azimayi amasiyira kumwa mankhwala? Kodi ndondomeko zaoteteza mwana kuti asatengere kachilombo ka HIV kuchokera kwa mayi (MPTCT) zingatani kuti zidzilimbikitsa amayi kuti adzibweleranso kuchipatala kukalandira chisamaliro ndi chithandizo cha kachilombo ka HIV?   1. M’maganizo mwanu, ndichisamaliro chanji chomwe mungaone kuti ndichopabana; chithandizo chopabana chomwe azaumoyo angapereke kwa amayi kapena amayi oyembekezera m’dera lino?   *[Funsisitsani]:* Mungapereke chitsanzo chinji chamu ndondomeko ya PMTCT chomwe chingafunikire kwenikweni m’dera lino?   1. Kodi m’dera lino, pali nkhwa ina ili yonse pazosungilana chinsinsi pa nkhani zikhudzana ndi kachilombo ka HIV? Kodi za kachilombo ka HIV zimakambidwa kwambiri?   *[Funsisitsani]:* Kodi vuto lenileni ndi chani ndipo mumathana nalo bwanji? Kodi mukaganiza kuti kusala anthu omwe ali ndi kachilombo ka HIV kwasinthilapo m’miyezi kapena zaka zingapo zapitazi? Ngati kwasinthilapo, kwasintha bwanji? Ngati sikunasinthe, mukuganiza kuti ndichifukwa chiyani?   1. M’mene mumaonera inuyo, kodi pali zipsinjo zomwe zimalepheretsa amayi ena omwe ali ndi kachilombo ka HIV kulandira chisamalilo cha PMTCT?   *[Funsisitsani]:* Mungafotokoze zipsyinjozi? Ndizofunikira bwanji m’dera lino? Ndichani chomwe chingathandize kuti anthu adzipeza chithandizo choyezetsa ndikulandira mankhwala?   1. Kodi pali zinthu zina zomwe zathandiza amayi ena omwe ali ndi kachilombo ka HIV kuti adzipeza chisamaliro ndi chithandizo m’dera lino?   *[Funsisitsani]:* Mungafotokoze zinthuzi? Zawathandiza bwanji amayi m’dera lino? |
| **Ndemanga zowonjezera**  *Tisanamalize kucheza muli ndi ndemanga ina iliyonse yokhudzana ndi nkhani yomwe takambilanayi? [Fotokozani]* |
| **Malangizo kwa ofunsa mafunso:**   - Athokozeni omwe mumacheza naowo chifukwa cha nthawi yawo ndipo akumbutseni kuti tidzafuna kucheza nawonso pakadutsa miyezi isanu ndi umodzi ngati angavomere. - Mukatha machezawo (tsiku lomwelo) lembani zomwe mumaona nthawi yamachezawo kuti zithandizile kufotokozela bwino zomwe mwapeza (mwachitsanzo malo, momwe machezawo amayendera, ngati panali kudulidwa, kukhudzidwa, kudodoma, maonekedwe a ofunsidwawo ndi zina zotero.) |

### Interview Guide for PMTCT Focal persons (Chichewa)

| **Ndondomeko yofunsira mafunso: otsogolera ndondomeko yoteteza mwana kuti asatengere kachilombo kuchokera kwa mayi**  Kafukufuka wa NEMAPP |
| --- |
| **Nambala ya**  **macheza: Boma:**  **Chipatala/Malo:**  **Tsiku lolowera mukafukufuku: (Tsiku) (Mwezi)**  **Nambala yamu kafukufuku**  **wa NEMAPP:**  **Zaka za ofunsidwa mafunso:**  **Ndiokwatiwa kapena**  **osakwatiwa:**  1= Okwatiwa  2= Nkazi wa mamasiye  3= Anasiyana  4= Sanakwatiwepo  5= China (longosoloni)________________________________  **Maphunziro**  1= Sanaphunzire  2= Makalasi am’munsi ku pulaimale  3= Makalasi a m’mwamba ku pulaimale  4= Makalasi a m’munsi ku sekondale  5= Makalasi a m’mwamba kusekondale  6= Adafika pa ukachenjede  7= Satifiketi kapena Dipuloma (longosolani)__________________________  8= China (longosolani)____________________________________________________  **Mtundu**  1= Muyao  2= Mulomwe  3= Mchewa  4= Mtumbuka  5= Mngoni  6= Msena  7= Wina (longosolani)________________________________  **Chipembedzo:**  1= Chikhilisitu  2= Chisilamu  3= Chikhalidwe  4 = Palibe kapena china (longosolani) ___________________________  **Mamuna/Nkazi:**  **Ofunsa mafunso/Otsogolera:**  **TSIKU/MWEZI/CHAKA**  **HRS**  **HRS**  **Tsiku: Nthawi yoyambira: Nthawi yomalizira:** |
| **Malangizo kwa ofunsa mafunso:**   - Tsalirani malamulo otengela chilolezo kuphatikizapo chilolezo chotenga macheza ndi chintapa mau. Mupatseni yemwe mukumufunsa mafunsoyo nthawi yokwanira yoti apangire chiganizo. Yemwe mukucheza nayeyo akhale omasuka kucheza nanu nthawi yopitilira ola limodzi (Ngati sangathe, pezani tsiku lina). - Mukuyenera kuwonetsetsa kuti chinsinsi chisungika (kuphatikizapo chinsinsi kuti zomwe zikulankhulidwa zisamveke) pa nthawi yomwe mukucheza. - Ili ndi dongosolo logwiritsidwa ntchito pa zokambirana zozama (osati pongofufuza pang’ono): mukuyenera kufunsa momwe mungathere pa zomwe wofunsidwayo wayankha kuti zimveke bwino ndipo funsisitsani mozama pa mutu uli onse. Machezawa adziyenda mooneka ngati mukungocheza osati ngati mukumufunsa chinthu munthu. - Kuchokera apa, machezawa atengedwe ndi chintapa mawu koma mukhale mutatenga chilolezo kuchokera kwa yemwe mukucheza nayeyo. - Lembani zomwe mukuona m’munsi mwa pepala lomweli patsiku lamacheza lomwelo. |
| 1. Maganizo anu ndi otani pachithandizo cha PMTCT chomwe chikuperekedwa pachipatala cha m’boma lino?   *[Funsisitsani]*: Zikuyenda bwino ndi ziti? Zikufunika zitasintha ndi ziti?   1. Mungafotokoze motani zimene azimayi amakumana nazo akamalandila chithandizo choperekedwa kwa azimayi kuti mwana obadwayo asatengere kachilombo (PMTCT) pachipatala chomwe anthu am’boma lino amapita?   *[Funsisitsani]*: Kodi amayi omwe amalandira chithandizo kudzera mu PMTCT anayamba akuuzanipo maganizo kapena awo nkhawa zawo?   1. Mumaganizo anu mukuwona kuti chikupangika ndi chani kapena chongamapangike ndi chani kuti azibambo adzitha kutenga nawo mbali polandira chithandizo choperekedwa kwa mayi kuti asapereke kachilombo kwa mwana? 2. Pali chithandizo china chimene inuyo mukuwona kuti chikusoweka? Pali chithandizo chilichonse chimene mukuwona kuti ndichofunika kwa amayi ndi mabanja awo? 3. Mukuyiwona bwanji ndondomeko yoyambitsira mankhwala otalikitsa moyo kwa odwala omwe ali ndi kachilombo ka HIV?   *[Funsisitsani]:* M’maganizo anu, ndichani chomwe chikuyenera kusinthidwa mundondomeko yoyambitsira mankhwala?   1. Mungafotokoze momwe mmaganizira kuti mau oti Option B+ amatanthauza chiyani?   *[Funsisitsani]:* Kuchokera momwe inu mukuonera, kodi ndondomekoyi imakhudza bwanji zokhudzana ndi chithandizo cha mankhwala a kachilombo ka HIV, kuyezetsa, kuyamwitsa ndi kuti abambo adzitengapo mbali?   1. Kodi mukuganiza kuti kulandila mankhwala otalikitsa moyo a ma ARV kungakhudze kuyamwitsa m’boma lino?   *[Funsisitsani]:* Zingakhudze bwanji kuyamwitsa ndipo zingakhudze kwambiri ndani?   1. Kodi mukuganiza kuti kulandila mankhwala otalikitsa moyo a ma ARV kungakhudze chifuniro cha mzimayi chofuna kudzakhalanso ndipakati m’boma lino?   *[Funsisitsani]:* Zingakhudze motani kukhala ndi pakati mtosgolo ndipo zingakhudze ndani kwambiri?   1. M’maganizo mwanu, mukuganiza kuti ndi chifukwa chiyani azimayi ena samabweranso kuchipatala kudzatenga mankhwala kapena samabweranso pa tsiku lomwe anawauza kuti abwerenso mzipatala m’boma lino?   *[Funsisitsani]:* M’mene mumawonera inu, ndizifukwa ziti zomwe azimayi amasiyira kumwa mankhwala? Kodi ndondomeko zaoteteza mwana kuti asatengere kachilombo ka HIV kuchokera kwa mayi (MPTCT) zingatani kuti zidzilimbikitsa amayi kuti adzibweleranso kuchipatala kukalandira chisamaliro ndi chithandizo cha kachilombo ka HIV?   1. M’maganizo mwanu, ndichisamaliro chanji chomwe mungaone kuti ndichopabana; chithandizo chopabana chomwe azaumoyo angapereke kwa amayi kapena amayi oyembekezera m’dera lino?   *[Funsisitsani]:* Mungapereke chitsanzo chinji chamu ndondomeko ya PMTCT chomwe chingafunikire kwenikweni m’boma lino?   1. Kodi m’boma lino, pali nkhwa ina ili yonse pazosungilana chinsinsi pa nkhani zikhudzana ndi kachilombo ka HIV? Kodi za kachilombo ka HIV zimakambidwa kwambiri?   *[Funsisitsani]:* Kodi vuto lenileni ndi chani ndipo mumathana nalo bwanji? Kodi mukaganiza kuti kusala anthu omwe ali ndi kachilombo ka HIV kwasinthilapo m’miyezi kapena zaka zingapo zapitazi? Ngati kwasinthilapo, kwasintha bwanji? Ngati sikunasinthe, mukuganiza kuti ndichifukwa chiyani?   1. M’mene mumaonera inuyo, kodi pali zipsinjo zomwe zimalepheretsa amayi ena omwe ali ndi kachilombo ka HIV kulandira chisamalilo cha PMTCT?   *[Funsisitsani]:* Mungafotokoze zipsyinjozi? Ndizofunikira bwanji m’boma lino? Ndichani chomwe chingathandize kuti anthu adzipeza chithandizo choyezetsa ndikulandira mankhwala?   1. Kodi pali zinthu zina zomwe zathandiza amayi ena omwe ali ndi kachilombo ka HIV kuti adzipeza chisamaliro ndi chithandizo m’boma lino?   *[Funsisitsani]:* Mungafotokoze zinthuzi? Zawathandiza bwanji amayi m’boma lino? |
| **Ndemanga zowonjezera**  *Tisanamalize kucheza muli ndi ndemanga ina iliyonse yokhudzana ndi nkhani yomwe takambilanayi? [Fotokozani]* |
| **Malangizo kwa ofunsa mafunso:**   - Athokozeni omwe mumacheza naowo chifukwa cha nthawi yawo ndipo akumbutseni kuti tidzafuna kucheza nawonso pakadutsa miyezi isanu ndi umodzi ngati angavomere. - Mukatha machezawo (tsiku lomwelo) lembani zomwe mumaona nthawi yamachezawo kuti zithandizile kufotokozela bwino zomwe mwapeza (mwachitsanzo malo, momwe machezawo amayendera, ngati panali kudulidwa, kukhudzidwa, kudodoma, maonekedwe a ofunsidwawo ndi zina zotero.) |

## Tumbuka Versions

### Interview Guide for Women (Tumbuka)

| **Mundondomeko wakendesyero kavidumbirano pakuchezga nabamama abo banamukhoza/nthumbo kweniso abo bakonkheska.**  Kafukufuku wa NEMAPP |
| --- |
| Kuchezga **#: Boma:**  **Chipatala/malo**  Zuba ilo mukanjirira mukafukufuku (Zuba) (Mwezi)    nambala yakafukufuku wa NEMAPP  **Vyaka vya uyo akuzgola:**  **Ngwakutengwa:**  1= ali panthengwa  2= mfumu wake alukufwa  3= balulekana nthengwa  4= bandatengwepo kawiro  5= vinyakhe (phalani)________________________________  **Masambiro**  1= Palije/wandayeko kusukulu  2= Wakalekera makalasi ghakusika ku primary  3= Wakalekera makalasi ghakuchanya ku primary  4= Wakalekera makalasi ghakusika ku secondary  5= Wakarekera makalasi ghakuchanya ku secondary  6= Walupanga masambiro ghapachanya/masambiro gha ukachenjede  7= Masatifiketi ghanyakhe namadipuloma (zunulani)____________________  8= Vinyakhe (zunulani)_______________________________________________  **Mutundu**  1= Muyao  2= Mulomwe  3= Muchewa  4= Mutumbuka  5= Mngoni  6= Musena  7= mutundu unyakhe (zunulani)______________________  **Chipembezo**  1= Mukhilisitu  2= Musilamu  3= Chabapapi/chaukhaliro  4= Palinje panji vinyakhe (zunulani)______________________  **Nambala yabana:**  **Sono ananthumbo/mukhoza**  1= Enya  2= Yayi  Bakokheska sono: Pala ni enya, kwamba pauli:  1= Enya  2= Yayi  **Wakufumba/wakwendeska vidumbirano:**    **ZUBA/MWEZI/CHILUMIKA**  **:**  **HRS**  **HRS**  **Zuba: Nyengo yakwambira: Nyengo yakumalira:** |
| **Vyakwenera kulondezga uyo wafumbenge mafumbo:**   - Londezgani mundondomeko wa chipepala chakulongola kuzomerezga kutolapo lwande mukafukufuku uyu, kweniso fumbani kuti bamuzomerezgani kujambula vidumbirano ivi. Mama uyo muchezgenge nayo uyo, mupasani nyengo yakukwanira kuti aghanaghane makola ghene. Munthu uyo muchezgenge nayo, abe wakufwasa/kusutuka pavidumbirano ivi ndipo muchezge kwa mphindi 60 (mungamanya kunozgaso ulendo unyakhe kuti muzakachezge nayo usange panyengo yasono nivyamachitiko yaye). - Ni udindo winu kuwonesyeska kuti kuchezga kwinu muvidumbirano ivi nikwamuchindindi/chisisi (kusazgirapo navyose ivyo vyajambulikanga pala mwadumbirananga). - Iyi nindondomeko yakendesyero kavidumbirano ivyo makani ghakhe pala muchezga mulongosolerana mwakufikapo khanira (nkhufubana waka mafumbo nopasana mazgoro yayi): fumbisiskani mwakufikapo pa mazgoro agho bakumuzgorani kuti bamuphalirani mwakupulikikwa makola ghene kweniso sandanisandani maghanoghano ghabo mwakufika khanira. Kuchezga kwinu kube nge mudumbirana munthu namunyakhe kuluska nakuti vibe nge mufubana waka mafumbo nopasana mazgoro nge nimumayeso. - Kufumira apa kuluta munthazi, kuchezga kwinu muvidumbirano ivi kujambulike, nachizomerezgo kufuma kwa abo mutichezgenge nabo. - Lembani ivyo mwaviwona kuti nivyakukhumbikwa/vyachandulo panyengo yeneyiyo kuumaliro kwa pepala ili, pazuba leneliro la kuchezga kwinu. |
| 1. *[Mafumbo ghakujimanyiska/kujiphala]: Apo titambengeko kuchezga kwithu muvidumbirano ivi muhawuno/zuba la lero, nakhumbanga kuti mujilongosole dani mwekha kuti kasi ndimwe banjani? Mulongosore vya umo mulili pa nyumba pinu?*   *[Fumbisiskani]: Ninjani uyo mukukhala nayo munyumba yinu? Mwakhala nyengo yitali wuli muchikaya chino? Mukugwira ntchito ulu? Mutemwa kuchita vichi usange kuti muli na nyengo yapawaka?*  *Nge umo nanguyoghoyera pawambiriro [ muvidumbirano vyakukhwafyana nachipepala chakulongora kuzomerezga kutorapo lwande mukafukufuku uyu], kwasono nitimufumbaninge mafumbo ghachoko pamakani ghakukhwafyana na HIV. Nkhugomezga kuti ivi viri makola kwa imwapa nakuti mulibakufwasa makola ghene pavidumbirano ivi. Mwakumukumbuskani waka kuti, usange panji mufwasenge yayi namafumbo ghanyakhe pavidumbirano ivi, muli banangwa kuleka kughazgora mafumbo nge ni agha ndipo tingamanya kulutilizga namafumbo ghakulondezga.*   1. Nikwa nyengo yitali ghuli iyo mwaba kuti mukuzakapokera wovyiri wavya HIV pachipatala chino (malo ghano)?   *[Fumbisiskani]: kasi iyi ni nyengo yinu yakwamba kuti mwize nakuzakapokera wovyira wakukhwafyana na HIV?*  *Panji mukalutangako kuchipatala chinyakhe kumasinda? Pala ninthena, nichipatala ghuli kweniso mukatola nyengo yitali ghuli mupokera wovyiri nge ni ughu kuchipatala chenechicho?*  *Nichifukwa ghuli mukaghanaghana vyakwiza kuchipatala chino?*   1. Chonde longosorani mutundu wabovyiri ugho mukupokera kwasono wakukhwafyana na HIV?   *[Fumbisiskani]: mupokerangaso ghovyiri unyakhe kufumira kwa babale binu, bafumu binu, bakugwira ntchito yavya umoyo, bamama bachisanzo, babwezi, panji banyakhe?*   1. Tiwerereko kumasinda panyengo iyo mukapimiskanga ndopa zinu pakuyana navya kachibungu ka HIV, pala muli bambula kukakika pamakani agha. Niphalirani vinandi vyakukhwafyana nanyengo iyo mukaba kuti mwapimiska waka ndopa zinu pakuyana navya kachibungu ka HIV?   *[Fumbisiskani]: chifukwa ghuli mukaghanaghana vyopimiska ndopa zinu? Bakamulimbikiskani kuti mukapimisye? Chikaba chisola chinu kuti mukapimisye? Mukakapimiska pakumoza namunthu munyakhe? Mukakapimiska nabafumu binu?*  *Mukachita ghuli/mukachipokerera ghuli apo mwati mwaphalirika vyakulondezga vyakupimiska ndopa zinu?*  *Mukaphalirapo njani vyakulondezga vyakupumiska ndopa zinu?*  *Nighovyiri ghuli ugho mukapokera panyengo iyo mukapimiska ndopa zinu, pala ungabapo?*   1. Mukafumbika/mukaphalirika kuti mwambeko kutola/kupokera ghovyiri wamunkhwala (ART) wa HIV?   *[Fumbisiskani]: nipaghuli apo mukafumbika/mukaphalirika kuti mwambeko kutola/kupokera ghovyiri wamunkhwala (ART) wa HIV?*  *Nivichi ivyo bakamuphalirani/longosolerani panyengo iyo mukati mwapimiska ndopa zinu?*  *Kasi ninyengo yitali ghuli iyo mubenge mutola/kupokera ghovyiri wamunkhwala ughu?*  *Kasi mukachipulika/mukachipokerera ghuli apo mukambanga kutola/kupokera ghovyiri wamunkhwala ughu?*  *Kasi mungapanga chisola chakwamba kupokera/kutola ghovyiri wamunkhwala ughu? [Fumbisiskani: kasi nimaghanoghano ghuli agho mukapanga panyengo iyo, kasi ntchivichi icho chikamupangiskani kuti mwize/mupange maghanoghano nge niagho?]*   1. Longosolani ivyo vikachitika mwati mwafumbika/mwaphalirika kuti mwambeko kutola/kupokera ghovyiri wamunkhwala (ART) wa HIV?   *[Fumbisiskani]: muludumbiranapo nabanjani pavyakukhwafyana naghovyiri wamunkhwala wa HIV?*  *Kasi pakatola nyengo yitali ghuli mwati mwapimiska ndopa zinu kuti mwambeko kutola/kupokera kakwamba ghovyiri wamunkwala wa HIV?*  *Kasi mukalutilizga kutola/kupokera ghovyiri wamunkhwala? Pala yayi, longosolani ivyo vikachitika [fumbisiskani pavifukwa ivyo bakalekera kulutilizga namunkhwala, ndipo bakaleka munkhwala paghuli, bakambiraso munkhwala, panyakhe bakalekeratu?]*   1. Kasi mukutola/mukumwa munkhwala mwakwenerera namundondomeko wakhe?   *[Fumbisiskani]: kasi mukuchipokerera/mukughaghana ghuli pavya munkhwala wa HIV?*  *Usange panji mutola cha/mukumwa yayi munkhwala nyengo zinyakhe, kasi nivifukwa ghuli ivyo mukulekeranga?*   1. Kasi mukabaphalira bafumu binu kuti mulupimiska ndopa zinu pakukhwafyana navya kachibungu ka HIV?   *[Fumbisiskani]: nanga pavya ghovyiri wamunkhwala wa HIV, kasi muludumbirana nabafumu binu?*  *Kasi bafumu binu balupimiska ndopa zabo pakukhwafyana nakachibungu ka HIV? Pala balupimiska, kasi balumuphalirani vyakulondezga vya umo ndopa zabo zilili bati bapimiska?*  *Kasi panji mumanya kuti bafumu binu bakupokera/bakumwa munkhwala wa HIV panyengo yasono?*  *Kasi mulugabanapo/kupasanapo nabafumu binu panji munthu munyakhe munkhwala wa HIV? [pala ni enya, fumbisiskani: ntchifukwa ghuli mukagabana/kupasana munkhwala, mukagabana/kupasana nabanjani, longosolani umo ivi vikachitikira, kasi kakaba kamoza pera panji vikichitikanga mwanyengo zose?]*  *Kasi mbanjani abo mulubaphalirapo/kudumbirana nabo vya umo mulili pakukhwafyana nakachibungu ka HIV? [Fumbisiskani: kasi mphaghuli apo mukabaphaliranga banthu aba? Mukalondezga mundondomeko ghuli apo mukabaphaliranga, kukaba kochi uko mukabaphaliranga, bakachipokerera ghuli apo mukati mwabaphalira?].*   1. Kasi mukapokera ghulangizi ghuli wakukhwafyana nakonkheska mwana?   *[Fumbisiskani]: kasi naumo mulukwambira kutola/kupokera ART, mulusithapo chilichose pakukhwafyana nakonkhesyero ka mwana winu?*  *[Lutilizgani kufumbisiska kuti mumanye pala kakhaliro panji kachitiro kakonkhesyero kalusitha kufumira apo bakambanga kutola/kumwa munkhwala (ART)]*   1. Kasi mungatemwa/mungakhumba kuzakatolaso mukhoza/nthumbo inyakhe kunthazi?   *[Fumbisiskani]: kasi kwamba kutola/kumwa munkhwala (ART) kulumupangiskani kuti muchitole mwakunyakhe pakukhwafyana navya kubaso namukhoza/nthumbo kunthazi?*  *[Lutilizgani kufumbisiska kuti mumanye pala munkhwala (ART) wiza nachandulo paumo vikwenera kubiranga pakuyana nauwemi panji uheni wakutola mukhoza/nthumbo]*   1. Mumaghanoghano ghinu, kasi palivinthu vinyakhe ivyo mukuvighona kuti vingachitikanga nachakulata cholutiska panthazi mundondomeko wakupimiska ndopa pakukhwafyana nakachibungu ka HIV kweniso kwambako kupokera/kumwa munkhwala wa HIV?   *[fumbisiskani]: mulibakufwasa kwambako kupokera/kumwa munkhwala wa HIV pala mwafuma waka popimiska ndopa zinu kukachibungu ka HIV?*  *Mumaghanoghano ghinu, kasi nighovyiri ghuli ugho ngwakwenerera kuperekeka kwa bamama namabanja ghabo nge lwande lumoza pachisamaliro cha HIV?* |
| **Vyakuyoghoya vyosazgirapo**  Pambere tindajale vidumbirano vithu, kasi muli nandemanga/vyakuyowoyapo virivyose vyakukhwafyana namakani agho tadumbirananga pakuchezga kwithu? [longosolani] |
| **Vyakwenera kuchita uyo ofumba mafumbo:**   - Bawongani abo bamuzgoraninge mafumbo/mwachezganga nabo chifukwa chakutola nyengo yabo nakutolapo lwande pakafukufuku uyu. Bakumbuskani bamama aba pala bangazomera, muzamukwizaso navidumbirano nge ni ivi pala pajumpha mwezi 6. - Pala mwamala vidumbirano vinu (zuba leneriro) lembani musika umu vyose ivyo mwavighonanga kuti vikayovyire kusazgirapo pa uthenga ugho watolereka pavidumbirano ivi (vingaba nge malo, umo vidumbirani vyayenderanga, vyakutimbanizga ivyo vyangubapo, umo kuchezga/mafumbo ghenderanga, umo angubira/apulikiranga pavidumbirano ivi, kuleka kufwasa/kujiwezga pakuyowoya pavidumbirano, umo alili ayo mwachezganga nayo, navinyakhe). |

### Interview Guide for LTFU women (Tumbuka)

| **Mundondomeko wakendesyero kavidumbirano: Babamama abo mba LTFU.**    Kafukufuku wa NEMAPP |
| --- |
| Kuchezga **#: Boma:**  **Chipatala/malo**  zuba ilo mukanjirira mukafukufuku (Muba) (Mwezi)    Nambala yakafukufuku wa NEMAPP  **Vyaka vya uyo akuzgola:**  **Ngwakutengwa:**  1= Ali panthengwa  2= Mfumu wake alukufwa  3= Balulekana nthengwa  4= Bandatengwepo kawiro  5= Vinyakhe (phalani)________________________________  **Masambiro**  1= Palije/wandayeko kusukulu  2= Wakalekera makalasi ghakusika ku primary  3= Wakalekera makalasi ghakuchanya ku primary  4= Wakalekera makalasi ghakusika ku secondary  5= Wakarekera makalasi ghakuchanya ku secondary  6= Walupanga masambiro ghapachanya/masambiro gha ukachenjede  7= Masatifiketi ghanyakhe namadipuloma (zunulani)____________________  8= Vinyakhe (zunulani)_______________________________________________  **Mutundu**  1= Muyao  2= Mulomwe  3= Muchewa  4= Mutumbuka  5= Mungoni  6= Musena  7= Mutundu unyakhe (zunulani)______________________  **Chipembezo**  1= Mukhilisitu  2= Musilamu  3= Chabapapi/chaukhaliro  4= Palinje panji vinyakhe (zunulani)______________________  **Nambala yabana:**  **Sono ananthumbo/mukhoza**  1= Enya  2= Yayi    Bakokheska sono: Pala ni enya, kwamba pauli:  1= Enya  2= Yayi  **Wakufumba/wakwendeska vidumbirano:**    **ZUBA/MWEZI/CHILUMIKA**    **HRS**  **HRS**  **Zuba: Nyengo yakwambira: Nyengo yakumalira:** |
| **Vyakwenera kulondezga uyo wafumbenge mafumbo:**   - Londezgani mundondomeko wa chipepala chakulongola kuzomerezga kutolapo lwande mukafukufuku uyu, kweniso fumbani kuti bamuzomerezgani kujambula vidumbirano ivi. Mama uyo muchezgenge nayo uyo, mupasani nyengo yakukwanira kuti aghanaghane makola ghene. Munthu uyo muchezgenge nayo, abe wakufwasa/kusutuka pavidumbirano ivi ndipo muchezge kwa mphindi 60 (mungamanya kunozgaso ulendo unyakhe kuti muzakachezge nayo usange panyengo yasono nivyamachitiko yaye). - Ni udindo winu kuwonesyeska kuti kuchezga kwinu muvidumbirano ivi nikwamuchindindi/chisisi (kusazgirapo navyose ivyo vyajambulika pala mwadumbirananga). - Iyi nindondomeko yakendesyero kavidumbirano ivyo makani ghakhe pala muchezga mulongosolerana mwakufikapo khanira (nkhufubana waka mafumbo nopasana mazgoro yayi): fumbisiskani mwakufikapo pa mazgoro agho bakumuzgorani kuti bamuphalirani mwakupulikikwa makola ghene kweniso sandanisandani maghanoghano ghabo mwakufika khanira. Kuchezga kwinu kube nge mudumbirana munthu namunyakhe kuluska nakuti vibe nge mufubana waka mafumbo nopasana mazgoro nge nimumayeso. - Kufumira apa kuluta munthazi, kuchezga kwinu muvidumbirano ivi kujambulike, nachizomerezgo kufuma kwa abo mutichezgenge nabo. - Lembani ivyo mwaviwona kuti nivyakukhumbikwa/vyachandulo panyengo yeneyiyo kuumaliro kwa pepala ili, pazuba leneliro la kuchezga kwinu. |
| 1. *[Mafumbo ghakujimanyiska/kujiphala]: Apo titambengeko kuchezga kwithu muvidumbirano ivi muhawuno/zuba la lero, nakhumbanga kuti mujilongosole dani mwekha kuti kasi ndimwe banjani? Mulongosore vya umo mulili pa nyumba pinu?*   *[Fumbisiskani]: Ninjani uyo mukukhala nayo munyumba yinu? Mwakhala nyengo yitali wuli muchikaya chino? Mukugwira ntchito ulu? Mutemwa kuchita vichi usange kuti muli na nyengo yapawaka?*  *Nge umo nanguyoghoyera pawambiriro [ muvidumbirano vyakukhwafyana nachipepala chakulongora kuzomerezga kutorapo lwande mukafukufuku uyu], kwasono nitimufumbaninge mafumbo ghachoko pamakani ghakukhwafyana na HIV. Nkhugomezga kuti ivi viri makola kwa imwapa nakuti mulibakufwasa makola ghene pavidumbirano ivi. Mwakumukumbuskani waka kuti, usange panji mufwasenge yayi namafumbo ghanyakhe pavidumbirano ivi, muli banangwa kuleka kughazgora mafumbo nge ni agha ndipo tingamanya kulutilizga namafumbo ghakulondezga.*   1. Vikughoneska/tamanya kuti mululeka kulutilizga/kupokera ghovyiri wakukhwafyana na HIV. Kasi nivifukwa ghuli ivyo vikamupangiskani kuti mughanaghane kuleka kulutilizga/kupokera ghovyiri wakukhwafyana na HIV?   *[Fumbisiskani]: longosolani vyaumo mukayighoneranga ntchito yopereka ghovyiri wakukhwafyana na HIV pachipatala icho mukalutangako?*  *Longosolani vyaumo mukaghoneranga ghovyiri wamunkhwala wa HIV?*   1. Kasi nikwa nyengo yitali ghuli iyo mukaba mupokera ghovyiri wakukhwafyana na HIV, pambere mukaba kuti mundaleke kwiza kuchipatala chino?   *[Fumbisiskani]: kasi yikaba nyengo yinu yakwamba kuti mupokere ghovyiri wa HIV?*  *Panji mukalutangako kuchipatala chinyakhe kumasinda? Pala ninthena, nichipatala ghuli kweniso mukatola nyengo yitali ghuli mupokera wovyiri nge ni ughu kuchipatala chenechicho?*   1. Chonde longosorani mutundu wabovyiri ugho mukupokera kwasono wakukhwafyana na HIV?   *[Fumbisiskani]: mupokerangaso ghovyiri unyakhe kufumira kwa babale binu, bafumu binu, bakugwira ntchito yavya umoyo, bamama bachisanzo, babwezi, panji banyakhe?*   1. Tiwerereko kumasinda panyengo iyo mukapimiskanga ndopa zinu pakuyana navya kachibungu ka HIV, pala muli bambula kukakika pamakani agha. Niphalirani vinandi vyakukhwafyana nanyengo iyo mukaba kuti mwapimiska waka ndopa zinu pakuyana navya kachibungu ka HIV?   *[Fumbisiskani]: chifukwa ghuli mukaghanaghana vyopimiska ndopa zinu? Bakamulimbikiskani kuti mukapimisye? Chikaba chisola chinu kuti mukapimisye? Mukakapimiska pakumoza namunthu munyakhe? Mukakapimiska nabafumu binu?*  *Mukachita ghuli/mukachipokerera ghuli apo mwati mwaphalirika vyakulondezga vyakupimiska ndopa zinu?*  *Mukaphalirapo njani vyakulondezga vyakupumiska ndopa zinu?*  *Nighovyiri ghuli ugho mukapokera panyengo iyo mukapimiska ndopa zinu, pala ungabapo?*   1. Mukafumbika/mukaphalirika kuti mwambeko kutola/kupokera ghovyiri wamunkhwala (ART) wa HIV?   *[Fumbisiskani]: nipaghuli apo mukafumbika/mukaphalirika kuti mwambeko kutola/kupokera ghovyiri wamunkhwala (ART) wa HIV?*  *Nivichi ivyo bakamuphalirani/longosolerani panyengo iyo mukati mwapimiska ndopa zinu?*  *Kasi ninyengo yitali ghuli iyo mubenge mutola/kupokera ghovyiri wamunkhwala ughu?*  *Kasi mukachipulika/mukachipokerera ghuli apo mukambanga kutola/kupokera ghovyiri wamunkhwala ughu?*  *Kasi mungapanga chisola chakwamba kupokera/kutola ghovyiri wamunkhwala ughu (ART)? [Fumbisiskani: kasi nimaghanoghano ghuli agho mukapanga panyengo iyo, kasi ntchivichi icho chikamupangiskani kuti mwize/mupange maghanoghano nge niagho?]*   1. Longosolani ivyo vikachitika mwati mwafumbika/mwaphalirika kuti mwambeko kutola/kupokera ghovyiri wamunkhwala (ART) wa HIV?   *[Fumbisiskani]: muludumbiranapo nabanjani pavyakukhwafyana naghovyiri wamunkhwala wa HIV?*  *Kasi pakatola nyengo yitali ghuli mwati mwapimiska ndopa zinu kuti mwambeko kutola/kupokera kakwamba ghovyiri wamunkwala wa HIV?*  *Kasi mukalutilizga kutola/kupokera ghovyiri wamunkhwala? Pala yayi, longosolani ivyo vikachitika [fumbisiskani pavifukwa ivyo bakalekera kulutilizga namunkhwala, ndipo bakaleka munkhwala paghuli, bakambiraso munkhwala, panyakhe bakalekeratu?]*   1. Vikughoneska/tamanya kuti mundawelerekoso kuchipatala pakanyengo sono. Kasi mukalutilizganga kupoka/kumwa munkhwala kwambula kuluta kuchipatala, panji mululekeratu?   *[Fumbisiskani]: pala mukalutilizganga kumwa/kupoka ART, kasi nikochi uko mukatolanga munkhwala?*  *kasi mukuchipokerera/mukughaghana ghuli pavya munkhwala wa HIV?*  *Usange panji mutola cha/mukumwa yayi munkhwala kwasono, kasi nivifukwa ghuli ivyo mulurekera?*   1. Kasi mukabaphalira bafumu binu kuti mulupimiska ndopa zinu pakukhwafyana navya kachibungu ka HIV?   *[Fumbisiskani]: nanga pavya ghovyiri wamunkhwala wa HIV, kasi muludumbirana nabafumu binu?*  *Kasi bafumu binu balupimiska ndopa zabo pakukhwafyana nakachibungu ka HIV? Pala balupimiska, kasi balumuphalirani vyakulondezga vya umo ndopa zabo zilili bati bapimiska?*  *Kasi panji mumanya kuti bafumu binu bakupokera/bakumwa munkhwala wa HIV panyengo yasono?*  *Kasi mulugabanapo/kupasanapo nabafumu binu panji munthu munyakhe munkhwala wa HIV? [pala ni enya, fumbisiskani: ntchifukwa ghuli mukagabana/kupasana munkhwala, mukagabana/kupasana nabanjani, longosolani umo ivi vikachitikira, kasi kakaba kamoza pera panji vikichitikanga mwanyengo zose?]*  *Kasi mbanjani abo mulubaphalirapo/kudumbirana nabo vya umo mulili pakukhwafyana nakachibungu ka HIV? [fumbisiskani: kasi mphaghuli apo mukabaphaliranga banthu aba? Mukalondezga mundondomeko ghuli apo mukabaphaliranga, kukaba kochi uko mukabaphaliranga, bakachipokerera ghuli apo mukati mwabaphalira?].*   1. Kasi mukapokera ghulangizi ghuli wakukhwafyana nakonkheska mwana?   *[Fumbisiskani]: kasi naumo mulukwambira kutola/kupokera ART, mulusithapo chilichose pakukhwafyana nakonkhesyero ka mwana winu?*  *[Lutilizgani kufumbisiska kuti mumanye pala kakhaliro panji kachitiro kakonkhesyero kalusitha kufumira apo bakambanga kutola/kumwa munkhwala (ART)]*   1. Kasi mungatemwa/mungakhumba kuzakatolaso mukhoza/nthumbo inyakhe kunthazi?   *[Fumbisiskani]: kasi kwamba kutola/kumwa munkhwala (ART) kulumupangiskani kuti muchitole mwakunyakhe pakukhwafyana navya kubaso namukhoza/nthumbo kunthazi?*  *[Lutilizgani kufumbisiska kuti mumanye pala munkhwala (ART) wiza nachandulo paumo vikwenera kubiranga pakuyana nauwemi panji uheni wakutola mukhoza/nthumbo]*   1. Mumaghanoghano ghinu, kasi palivinthu vinyakhe ivyo mukuvighona kuti vingachitikanga nachakulata cholutiska panthazi mundondomeko wakupimiska ndopa pakukhwafyana nakachibungu ka HIV kweniso kwambako kupokera/kumwa munkhwala wa HIV?   *[Fumbisiskani]: mulibakufwasa kwambako kupokera/kumwa munkhwala wa HIV pala mwafuma waka popimiska ndopa zinu kukachibungu ka HIV?*  *Mumaghanoghano ghinu, kasi nighovyiri ghuli ugho ngwakwenerera kuperekeka kwa bamama namabanja ghabo nge lwande lumoza pachisamaliro cha HIV?* |
| **Vyakuyoghoya vyosazgirapo**  Pambere tindajale vidumbirano vithu, kasi muli nandemanga/vyakuyowoyapo virivyose vyakukhwafyana namakani agho tadumbirananga pakuchezga kwithu? [Longosolani] |
| **Vyakwenera kuchita uyo ofumba mafumbo:**   - Bawongani abo bamuzgoraninge mafumbo/mwachezganga nabo chifukwa chakutola nyengo yabo nakutolapo lwande pakafukufuku uyu. Bakumbuskani bamama aba kuti bangazomera, muzamukwizaso navidumbirano nge ni ivi pala pajumpha mwezi 6. - Pala mwamala vidumbirano vinu (zuba leneriro) lembani musika umu vyose ivyo mwavighonanga kuti vikayovyire kusazgirapo pa uthenga ugho watolereka pavidumbirano ivi (vingaba nge malo, umo vidumbirano vyayenderanga, vyakutimbanizga ivyo vyangubapo, umo kuchezga/mafumbo ghenderanga, umo angubira/apulikiranga pavidumbirano ivi, kuleka kufwasa/kujiwezga pakuyowoya pavidumbirano, umo alili uyo mwachezganga nayo, navinyakhe). |

### Focus group discussion guide for Health care workers (Tumbuka)

| **Mundondomeko wakendesyero kavidumbirano vya pagulu pakuchezga nabakugwira ntchito yavya umoyo (chomenechomene bakugwira ntchito yavya option B+).**  Kafukufuku wa NEMAPP | | | | |
| --- | --- | --- | --- | --- |
| FGD**#: Boma:**  **Chipatala:**  **AM/PM**  **AM/PM**  Zuba: Nyengo yakwambira: Nyengo yakumalira: | | | | |
|  | **Vyaka** | **Mwanalume/**  **mwanakazi** | **Masambiro** | **Ntchito yaupangiri (nesi, HSA, dokotala, zinyankhe zunurani)** |
| 1 |  |  |  |  |
| 2 |  |  |  |  |
| 3 |  |  |  |  |
| 4 |  |  |  |  |
| 5 |  |  |  |  |
| 6 |  |  |  |  |
| 7 |  |  |  |  |
| 8 |  |  |  |  |
| 9 |  |  |  |  |
| 10 |  |  |  |  |
| **Masambiro:**  1= Palije/wandayeko kusukulu, 2= Wakalekera makalasi ghakusika ku primary, 3= Wakalekera makalasi ghakuchanya ku primary, 4= Wakalekera makalasi ghakusika ku secondary, 5= Wakarekera makalasi ghakuchanya, ku secondary, 6= Walupanga masambiro ghapachanya/masambiro gha ukachenjede  7= Masatifiketi ghanyakhe namadipuloma (zunulani), 8= Vinyakhe (zunulani)  **Wakwendeska vidumbirano vyapa gulu (FGD):** | | | | |
| **Vyakwenera kulondezga uyo wayendesyenge vidumbirano vyapa gulu (FGD):**   - Ndondomeko iyi niyakuti yimovyirani pakwambiska nakwendeska vidumbirano vyapa gulu, ivyo vibenge vyakuti nge mukudumbirana munthu namunyakhe kuluska nakuti vibe nge mufubana waka mafumbo nopasana mazgoro nge nimumayeso. - Ndondomeko iyi ilupangika kuyendeska vidumbirano vya gulu labanthu 4-6 abo bakugwira ntchito yavya umoyo, pachipatala chilichose pabenge pachitikenge kamoza pera vidumbirano vyapa gulu. Nikwakukhumbikwa chomene kuti muvidumbirano vyapagulu nge ni ivi mubemo banalume na banakazi. - Abo bakutolapo lwande muvidumbirano vyapa gulu ivi bakwenerera kumanyiskika kuti vidumbirano ivi vitorenge mphindi 60 (nozgani nyengo inyakhe pala kwasono nivyamachitiko yayi). - Fumbani yumoza wamamembala gha abo bakwendeska kafukufuku uyu kuti alembenge vyose mwakuyana na umo vidumbirano vikwendera mwakukhwafyana na umo banthu bakuyoghoyesherana, umo gulu likwendera pavidumbirano, kweniso namakani ghose umo ghakwendera muvidumbirano ivi. - Ghonesyeskani kuti pavidumbirano ivi mwasunga chisisi (kusungaso chisisi cha vidumbirano ivyo mwajambula). Abo bakutolapo lwande pakafukufuku uyu mbakuti panji bakumanyana kale, theula mupange malamulo nachakulata chakuti ivyo mudumbiranenge nge ni gulu vibe vya muchindindi/chisisi. | | | | |
| Ise tukhumba tidumbirane namwe na umo ntchito ya chipwelerero cha PMTCT yikuchitikira kweniso tipulike vya ivyo mukuvighona naumo zintchito za zuba na zuba zilili pachipatala chino.   1. Kasi maghanoghano ghinu ghakutighuli pachipwelerero cha PMTCT icho chuperekeka pachipatala chino, ivyo vikwenda makola nivichi kweniso ivyo vikwenera kusitha nivichi? 2. Kasi mungalongosolapo vichi pa ivyo bakusangana navyo bamama abo bakupokera ghovyiri wachipwelerero cha PMTCT pachipatala chino?      1. Mumaghanoghano ghinu, kasi nivichi ivyo vikuchitika panji vingachitikanga nachakulata chakuti badada/banalume nabo batolengepo lwande pakupokera chipwelerero cha ntchito ya PMTCT? 2. Kasi ghulipo ghovyiri ghulighose ugho mukughuwona kuti ghukusobekera? Panji pali ghovyiri wakukhumbikwa kwa bamama namabanja ghabo kuti uperekekenge pachipatala pano? 3. Kasi mukughughona ghuli mundondomeko na umo ulili/ukwendera pala bakwambiska balwali pa munkhwala/chipwelerero cha ART?   Mumaghanoghano ghinu, kasi nivichi ivyo vikwenera kusitha pa mundondomeko ugho bakulondezga pakumwambiska munthu munkhwala?     1. Longosolani na umo mukuyighonera ntchito ya Option B+?     Kasi pulogilamu iyi yikusithangapo vichi pamakani ghakukhwafyana na ghovyiri wamunkhwala wa HIV, kupimiska ndopa, kokheska bana kweniso pamakani ghakulimbikiska badada/banalume kutolapo lwande pa PMTCT?   1. Mumaghanoghano ghinu, kasi nichifukwa ghuli mukughaghana kuti bamama banyakhe balekera panthowa kumwa munkhwala wa HIV (LTFU – Lost To Follow Up)?   Kasi nivifukwa ghuli bamama banyakhe bakulekeranga panthowa kumwa munkhwala wa HIV (LTFU), mukughona kwinu? Kasi chipwelerero cha PMTCT chingachepeska ghuli chiberengero chabamama abo bakulekera panthowa kumwa munkhwala wa HIV (LTFU)?   1. Mumaghanoghano ghinu, kasi mukughona nge nighovyiri ghuli wapachanya ugho ungapasikanga kwa bamama abo banamukhoza/nthumbo kweniso bakokheska pakukhwafyana namundondomeko wa PMTCT? Kasi nintchito ghuli yavya umoyo yakukhumbikwa chomene iyo chipatala chino chikuchita panyengo yasono? (longosolani). | | | | |
| **Vyakwenera kulondezga uyo wayendesyenge vidumbirano vyapa gulu (FGD):**     - Bawongani banthu bose abo mwachezganga nabo chifukwa chakupereka nyengo yabo nakutolapo lwande pakafukufuku uyu kweniso balongosolerani kuti muzamukwizaso navidumbirano vyapa gulu nge ni ivi pala pajumpha mwezi 6. - Zuba leneriro, lembani vyose ivyo mwavighonanga pala mwamala waka vidumbirano vyapa gulu ivi, lembani chilichose chakukhwafyana naumo gulu ili layenderanga pavidumbirano ivi, vyakutimbanizga kweniso chilichose icho ntchakukhumbikwa kuti vikayovyire kusazgirapo pa uthenga ugho watolereka pavidumbirano ivi. | | | | |

### Interview Guide for Male Partners (Tumbuka)

| **Mundondomeko wakendesyero kavidumbirano pakuchezga nabadada abo bawoli babo banamukhoza/nthumbo kweniso abo bakonkheska.**    KafukufukU wa NEMAPP |
| --- |
| Kuchezga **#: Boma:**  **Chipatala/malo**  Zuba ilo mukanjirira mukafukufuku (Zuba) (Mwezi)  Nambala yakafukufuku wa NEMAPP  **Vyaka vya uyo akuzgola:**  **Ngwakutengwa:**  1= Ali panthengwa  2= Mfumu wake alukufwa  3= Balulekana nthengwa  4= Bandatengwepo kawiro  5= Vinyakhe (phalani)________________________________  **Masambiro**  1= Palije/wandayeko kusukulu  2= Wakalekera makalasi ghakusika ku primary  3= Wakalekera makalasi ghakuchanya ku primary  4= Wakalekera makalasi ghakusika ku secondary  5= Wakarekera makalasi ghakuchanya ku secondary  6= Walupanga masambiro ghapachanya/masambiro gha ukachenjede  7= Masatifiketi ghanyakhe namadipuloma (zunulani)____________________  8=vinyakhe (zunulani)_______________________________________________  **Mutundu**  1= Muyao  2= Mulomwe  3= Muchewa  4= Mutumbuka  5= Mungoni  6= Musena  7= Mutundu unyakhe (zunulani)______________________  **chipembezo**  1= Mukhilisitu  2= Musilamu  3= Chabapapi/chaukhaliro  4= Palinje panji vinyakhe (zunulani)______________________  **Nambala yabana:**  **Sono ananthumbo/mukhoza**  1= Enya  2= Yayi    Bakokheska sono: Pala ni enya, kwamba pauli:  1= Enya  2= Yayi  **Wakufumba/wakwendeska vidumbirano:**    **ZUBA/MWEZI/CHILUMIKA**    **HRS**  **HRS**  **Zuba: Nyengo yakwambira: Nyengo yakumalira:** |
| **Vyakwenera kulondezga uyo wafumbenge mafumbo:**   - Londezgani mundondomeko wa chipepala chakulongola kuzomerezga kutolapo lwande mukafukufuku uyu, kweniso fumbani kuti bamuzomerezgani kujambula vidumbirano ivi. Mama uyo muchezgenge nayo uyo, mupasani nyengo yakukwanira kuti aghanaghane makola ghene. Munthu uyo muchezgenge nayo, abe wakufwasa/kusutuka pavidumbirano ivi ndipo muchezge kwa mphindi 60 (mungamanya kunozgaso ulendo unyakhe kuti muzakachezge nayo usange panyengo yasono nivyamachitiko yaye). - Ni udindo winu kuwonesyeska kuti kuchezga kwinu muvidumbirano ivi nikwamuchindindi/chisisi (kusazgirapo navyose ivyo vyajambulika pala mwadumbirananga). - Iyi nindondomeko yakendesyero kavidumbirano ivyo makani ghakhe pala muchezga mulongosolerana mwakufikapo khanira (nkhufubana waka mafumbo nopasana mazgoro yayi): fumbisiskani mwakufikapo pa mazgoro agho bakumuzgorani kuti bamuphalirani mwakupulikikwa makola ghene kweniso sandanisandani maghanoghano ghabo mwakufika khanira. Kuchezga kwinu kube nge mudumbirana munthu namunyakhe kuluska nakuti vibe nge mufubana waka mafumbo nopasana mazgoro nge nimumayeso. - Kufumira apa kuluta munthazi, kuchezga kwinu muvidumbirano ivi kujambulike, nachizomerezgo kufuma kwa abo mutichezgenge nabo. - Lembani ivyo mwaviwona kuti nivyakukhumbikwa/vyachandulo panyengo yeneyiyo kuumaliro kwa pepala ili, pazuba leneliro la kuchezga kwinu. |
| 1. Kasi panyengo yasono mukupokera ghovyiri ghulighose pachipatala icho bawoli binu bakulutangako kukapokera ghovyiri? [fumbisiskani, nighovyiri ghuli]. 2. Nilongosolerani pala mulupimiskapo ndopa zinu kuti mumanye kuti mulinako panji mulije kachibungu ka HIV? Kweniso longosolani pala mulupokerapo ghovyiri ghulighose apo mukaba muchali mundapimisye ndopa zinu panji apo mukati mwapimiska ndopa zinu? 3. Longosolani vya ghovyiri ugho bawoli banu bakupokera kwa sono wakukhwafyana na HIV? **[fumbisiskani kuti nighovyiri ghuli ugho bakupokera kufumira kubale panyumba, kwa ibo nge wakutemweka wabo/fumu wabo, bakugwira ntchito yavya umoyo, balangizi ba bamama abo bali nakachibungu ka HIV, babwezi babo, nabanyakhe].** 4. Kasi ghovyiri ugho bawoli binu bakupokera kwasono wakukhwafyana na HIV, kusazgirapo ghovyiri wamunkhwala wa HIV, ghukulekana na maghovyiri ghanyakhe agho ghakuperekekanga pachipatala chino? [**pala ngwakulekana, ukulekana ghuli? Chonde longosolani].** 5. Kasi mukughanaghana kuti munkhwala ugho bawoli binu bakupokera ungamanyaso kukhuza imwapa pa umoyo winu? **[fumbisiskani kuti ungakhuza ghuli moyo wabo]** 6. Kasi imwapa mukupokera ghovyiri wakuyana waka na ugho bakupokera bagholi binu kwasono? [**Fumbisiskani kuti ukuyana panji ukulekana ghuli ghovyiri].** |
| 1. ***[pala balupimiskapo ndopa zabo pakukhwafyana na HIV]:*** niphalirani vinandi vyakuyana na nyengo iyo mukati mwapimiska ndipa zinu pakukhwafyana na HIV? Fumbisiskani kuti ntchifukwa ghuli bakakapimiska ndopa zawo? Mukachipokerera ghuli mwati mwaphalirika vya kulondezga vya kumiska kwa ndopa zinu? Mukakaphalirapo njani vyakulondezga vya kupimiska kwa ndopa zinu? Kasi mukapokera ghovyiri ghuli pa nyengo iyo mukati mwapimiska ndopa zinu?]. 2. ***[pala balupimiskapo ndopa zabo nakusangika kuti bali nakachibungu ka HIV]:*** nikwamba pa uli apo mukaphalirika/mukafumbika kuti mwambeko kumwa munkhwala wakutalikiska moyo (ma ARV)? [fumbisiskani kuti kasi pakatola nyengo yitali ghuli apo bati bapimiska ndopa zabo pakukhwafyana na HIV kuti bambeko kumwa munkhwala wakutalikiska moyo (ma ARV)?] 3. ***[pala bakumwa munkhwala wakutalikiska moyo (ma ARV)]: nilongosolerani pa ivyo mwaba musangana navyo kufumira apo mukati mwapimiska waka ndopa zinu pakukhwafyana na HIV kuzakafika kuti mwambako kumwa munkhwala wakutalikiska moyo (ma ARV), kuzakafika mpakana sono?*** 4. ***[pala bakumwa munkhwala wakutalikiska moyo (ma ARV)]:*** kasi mukumwa munkhwala wama ARV munyengo yake yakubikika? [fumbisiskani pa vya kusuzga ivyo bakusangana navyo pala bakumwa munkhwala wakutalikiska moyo wama ARV]. 5. Kasi mukumanya vya masuzgo agho bawoli binu bakusangana nabo apo bakupokera/bakumwa munkhwala wakutalikiska moyo wama ARV? [fumbisiskani kuti kasi numasuzgo ghuli]. 6. Kasi mulinagho maghanoghano ghozakababaso bana banyakhe munthazi muno? [fumbisiskani: longosolani vifukwa ivyo vikumupangiskani kuti muzakababeso bana panji muleke kuzakababaso bana banyakhe munthazi muno? Kasi mukughanaghana kuti apo bawoli binu bakumwa munkhwala wakutalikiska moyo wama ARV vikukhwafyanapo mulimose nakuzakababaso bana banyakhe munthazi? Vikukhwafyana ghuli? 7. Mumaghanoghano ghinu, kasi palivinthu vinyakhe ivyo mukuvighona kuti vingachitikanga nachakulata cholutiska panthazi mundondomeko wakupimiska ndopa pakukhwafyana nakachibungu ka HIV kweniso kwambako kupokera/kumwa munkhwala wa HIV? 8. Kasi mulukumanapo nabanalume abo bakukana kupimiska ndopa zabo pakukhwafyana na HIV panyakhe bakukana kwambako kumwa munkhwala wakutalikiska moyo wama ARV? [fumbisiskani: longosolani vufukwa ivyo bakukanira]. 9. Mumaghanoghano ghinu, kasi nighovyiri ghuli ugho ngwakwenerera kuperekeka kwa bamama namabanja ghabo nge lwande lumoza pachisamaliro cha HIV? |
| 1. ***[pala balupimiskapo ndopa zabo pakukhwafyana na HIV]:*** *Kasi mbanjani abo mulubaphalirapo/kudumbirana nabo vya umo mulili pakukhwafyana nakachibungu ka HIV? [fumbisiskani: kasi mphaghuli apo mukabaphaliranga banthu aba? Mukalondezga mundondomeko ghuli apo mukabaphaliranga, kukaba kochi uko mukabaphaliranga, bakachipokerera ghuli apo mukati mwabaphalira?].* 2. ***[pala balupimiskapo ndopa zabo pakukhwafyana na HIV]:*** *Mukalondezga mundondomeko ghuli apo mukabaphaliranga, kukaba kochi uko mukabaphaliranga, bakachipokerera ghuli apo mukati mwabaphalira?* 3. ***[pala balupimiskapo ndopa zabo pakukhwafyana na HIV]:****.* Kasi mukabaphalira bawoli binu kuti mulupimiska ndopa zinu pakukhwafyana navya kachibungu ka HIV? [*pala yayi, fumbani vifukwa vyakhe/ pala enya, fumbani mundondomeko ugho bakalondezga apo bakabaphaliranga bawoli babo].* 4. Mukughanaghana kuti ntchakukhumbikwira kuti munthu aghulurenge ku banthu vya umo alili usange apimiska ndopa zake pakukhwafyana nakabungu ka HIV? Chifukwa ghuli? [fumbisiskani kuti nivyakusuzga ghuli ivyo angasangana navyo pala angaphalira banthu vya umo munthupi mwake alili pakukhwafyana navya kupimiska ndopa nakachibungu ka HIV]. |
| Pambere tindajale vidumbirano vithu, kasi muli nandemanga/vyakuyowoyapo virivyose vyakukhwafyana namakani agho tadumbirananga pakuchezga kwithu? [longosolani] |
| **Vyakwenera kuchita uyo ofumba mafumbo:**   - Bawongani abo bamuzgoraninge mafumbo/mwachezganga nabo chifukwa chakutola nyengo yabo nakutolapo lwande pakafukufuku uyu. Kweniso baphalirani kuti muzamukwizaso navidimbira nge ni ivi pala pajumpha mwezi 6. - Pala mwamala vidumbirano vinu (zuba leneriro) lembani musika umu vyose ivyo mwavighonanga kuti vikayovyire kusazgirapo pa uthenga ugho watolereka pavidumbirano ivi (vingaba nge malo, umo vidumbirano vyayenderanga, vyakutimbanizga ivyo vyangubapo, umo kuchezga/mafumbo ghenderanga, umo angubira/apulikiranga pavidumbirano ivi, kuleka kufwasa/kujiwezga pakuyowoya pavidumbirano, umo alili uyo mwachezganga nayo, navinyakhe). |

### Interview Guide for Community Leaders (Tumbuka)

| **Mundondomeko wakendesyero kavidumbirano pakuchezga: Babalalabalala bamuvikaya nge mafumu.**  Kafukufuku wa NEMAPP |
| --- |
| Kuchezga **#: Boma:**  **Chipatala/malo**  Zuba ilo mukanjirira mukafukufuku (Zuba) (Mwezi)    Nambala yakafukufuku wa NEMAPP  **Vyaka vya uyo akuzgola:**  **Ngwakutengwa:**  1= ali panthengwa  2= mfumu wake alukufwa  3= balulekana nthengwa  4= bandatengwepo kawiro  5= vinyakhe (phalani)________________________________  **Masambiro**  1= Palije/wandayeko kusukulu  2= Wakalekera makalasi ghakusika ku primary  3= Wakalekera makalasi ghakuchanya ku primary  4= Wakalekera makalasi ghakusika ku secondary  5= Wakarekera makalasi ghakuchanya ku secondary  6= Walupanga masambiro ghapachanya/masambiro gha ukachenjede  7= Masatifiketi ghanyakhe namadipuloma (zunulani)____________________  8= Vinyakhe (zunulani)_______________________________________________  **Mutundu**  1= Muyao  2= Mulomwe  3= Muchewa  4= Mutumbuka  5= Mngoni  6= Musena  7= mutundu unyakhe (zunulani)______________________  **Chipembezo**  1= Mukhilisitu  2= Musilamu  3= Chabapapi/chaukhaliro  4= Palinje panji vinyakhe (zunulani)______________________  **Mwanalume/Mwanakazi:**  **Wakufumba/wakwendeska vidumbirano:**    **ZUBA/MWEZI/CHILUMIKA**    **HRS**  **HRS**  **Zuba: Nyengo yakwambira: Nyengo yakumalira:** |
| **Vyakwenera kulondezga uyo wafumbenge mafumbo:**   - Londezgani mundondomeko wa chipepala chakulongola kuzomerezga kutolapo lwande mukafukufuku uyu, kweniso fumbani kuti bamuzomerezgani kujambula vidumbirano ivi. Mama uyo muchezgenge nayo uyo, mupasani nyengo yakukwanira kuti aghanaghane makola ghene. Munthu uyo muchezgenge nayo, abe wakufwasa/kusutuka pavidumbirano ivi ndipo muchezge kwa mphindi 60 (mungamanya kunozgaso ulendo unyakhe kuti muzakachezge nayo usange panyengo yasono nivyamachitiko yaye). - Ni udindo winu kuwonesyeska kuti kuchezga kwinu muvidumbirano ivi nikwamuchindindi/chisisi (kusazgirapo navyose ivyo vyajambulikanga pala mwadumbirananga). - Iyi nindondomeko yakendesyero kavidumbirano ivyo makani ghakhe pala muchezga mulongosolerana mwakufikapo khanira (nkhufubana waka mafumbo nopasana mazgoro yayi): fumbisiskani mwakufikapo pa mazgoro agho bakumuzgorani kuti bamuphalirani mwakupulikikwa makola ghene kweniso sandanisandani maghanoghano ghabo mwakufika khanira. Kuchezga kwinu kube nge mudumbirana munthu namunyakhe kuluska nakuti vibe nge mufubana waka mafumbo nopasana mazgoro nge nimumayeso. - Kufumira apa kuluta munthazi, kuchezga kwinu muvidumbirano ivi kujambulike, nachizomerezgo kufuma kwa abo mutichezgenge nabo. - Lembani ivyo mwaviwona kuti nivyakukhumbikwa/vyachandulo panyengo yeneyiyo kuumaliro kwa pepala ili, pazuba leneliro la kuchezga kwinu. |
| 1. Kasi maghanoghano ghinu ghakuti ghuli pachipwelerero cha PMTCT icho chuperekeka pachipatala chino?   *[Fumbiskani]*: Ivyo vikwenda makola nivichi kweniso ivyo vikwenera kusitha nivichi?   1. Kasi mungalongosolapo ivyo bakusangana navyo bamama abo bakupokera ghovyiri wachipwelerero cha PMTCT pachipatala chino?   *[Fumbiskani]:* Kasi banakazi aba bakumumanyani kweniso abo bakutorapo chipwererero cha PMTCT, bamuphaliranipo mazgo ghakudandaura panyake masuzgo?   1. Mumaghanoghano ghinu, kasi nivichi ivyo vikuchitika panji vingachitikanga nachakulata chakuti badada/banalume nabo batolengepo lwande kuvipwelerero ivyi? 2. Kasi ghulipo ghovyiri ghulighose ugho mukughuwona kuti ghukusobekera? Panji pali ghovyiri wakukhumbikwa kwa bamama namabanja ghabo kuti uperekekenge pachipatala pano? 3. Kasi mukughughona ghuli mundondomeko na umo ulili/ukwendera pala bakwambiska balwali pa munkhwala wa HIV, chomenechomene kwa bamama abo bakupokera ghovyiri wa PMTCT?   *[Fumbisiskani]:* Mumaghanoghano ghinu, kasi nivichi ivyo vikwenera kusitha pa mundondomeko ugho bakulondezga pakumwambiska munthu munkhwala?     1. Mazgu gha ‘Option B+’ mukubamanya? Para mukubamanya, mukugomezga kuti ghakung’anamura vichi?   *[Fumbisiskani]:* Kasi pulogilamu iyi yikusithangapo vichi pamakani ghakukhwafyana na ghovyiri wamunkhwala wa HIV, kupimiska ndopa, kokheska bana kweniso pamakani ghakulimbikiska badada/banalume kutolapo lwande pa PMTCT?   1. Kasi mukuyanayana kuti kuba paghovyiri wa munkhwala wa HIV kungiza nachandulo chilichose pamakani ghakokheska bana muchikaya/mumuzi muno?   *[Fumbisiskani]: pangaba chandulo ghuli pamakani ghakokheska bana pakuyana nakuba paghovyiri wakupokera munkhwala wa HIV, ndipo ninjani uyo ukukhwafyika chomene?*   1. Kasi mughanaghana kuti kuba poghovyiri wamunkhwala wa ART kungaba nachandulo kuti bamama babe namaghanoghano ghakutolaso nthumbo zinyakhe muchikaya/mumuzi muno?   *[Fumbisiskani]: kasi pangaba chandulo ghuli pamakani ghakutolaso nthumbo inyakhe pakuyana nakuba paghovyiri wakupokera munkhwala wa ART, ndipo ninjani uyo ukukhwafyika chomene?*   1. Mumaghanoghano ghinu, kasi mukughanaghana kuti nivifukwa ghuli ivyo vikupangiska bamama kuleka kwizaso kuzakatola munkhwala panji kwizaso pa zuba labo lakubikika kuno kuchipatala?   *[Fumbisiskani]: mukuwona kwinu, nivifukwa ghuli ivyo bamama bakulekera kumwa munkhwala? Kasi mundondomeko wa chipwererero cha PMTCT, wendesyekenge ghuli kuti bamama balimbiskike kwizaso kuchipatala kuzakapoka ghovyiri wamunkhwala nachipwererero cha HIV?*   1. Mumaghanoghano ghinu, kasi mukughona nge nighovyiri ghuli wapachanya ugho ungapasikanga kwa bamama panji abo banamukhoza/nthumbo muchikaya/ mumuzi muno?     *[Fumbisiskani]: tiphalirani umoza mwa maghovyiri pakuyana nachiphwererero cha PMTCT agho ghangakhumbikwa kuperekekanga muchikaya/mumuzi muno?*   1. Muchikaya chinu/muzi winu, kasi makani ghakukhwafyana na HIV ghakubikika kuba ghamuchindindi/chisisi? Kasi makani gha HIV ghakuyoghoyekanga mwakufikapo?   *[Fumbisiskani]: kasi chomenechomene mukuphwerererapo makani ghuli ghakukhwafyana na HIV ndpo mukumalananga nagho ghuli makani nge ni agha? Kasi mu mwezi panji vyaka vyajumpha ivi, mukughanaghana kuti pali kusitha kulikose pakuyana nakugegana pakukhwafyana nabanthu abo bali nakachibungu ka HIV? Pala kulipo kusitha kulikose, chonde phalani. Pala palije kusitha kulikose, longosolani kuti kasi nichifukwa ghuli palije kusitha.*   1. Mukuwona kwinu, kasi vilipo vyakujanda vilivyose ivyo vingapangiska bamama abo bali nakachibungu ka HIV kuba poghovyiri wa chiphwererero cha PMTCT?   *[Fumbisiskani]:* longosolani vyakujanda pala viripo? Kasi viri nachandulo ghuli muchikaya/muzi ghuno? Kasi nivichi ivyo vingalutiska panthazi mulimo/ntchito yakupimiska ndopa pakukhwafyana nakachibungu ka HIV naghovyiri wamunkhwala?   1. Kasi nizifundo ghuli izo ziluvyirapo bamama abo bali nakachibungu ka HIV muchikaya chino/muzi ghuno kupokera ghovyiri wamunkhwala nachiphwererero pakukhwafyana na HIV?   *[Fumbisiskani]: longosolani zifundo izi pala zilipo? Kasi zilupindulirapo ghuli bamama mumuzi panji chikaya chino?* |
| Pambere tindajale vidumbirano vithu, kasi muli nandemanga/vyakuyowoyapo virivyose vyakukhwafyana namakani agho tadumbirananga pakuchezga kwithu? [longosolani] |
| **Vyakwenera kuchita uyo ofumba mafumbo:**   - Bawongani abo bamuzgoraninge mafumbo/mwachezganga nabo chifukwa chakutola nyengo yabo nakutolapo lwande pakafukufuku uyu. Bakumbuskani bamama aba kuti bangazomera, muzamukwizaso navidumbirano nge ni ivi pala pajumpha mwezi 6. - Pala mwamala vidumbirano vinu (zuba leneriro) lembani musika umu vyose ivyo mwavighonanga kuti vikayovyire kusazgirapo pa uthenga ugho watolereka pavidumbirano ivi (vingaba nge malo, umo vidumbirano vyayenderanga, vyakutimbanizga ivyo vyangubapo, umo kuchezga/mafumbo ghenderanga, umo angubira/apulikiranga pavidumbirano ivi, kuleka kufwasa/kujiwezga pakuyowoya pavidumbirano, umo alili uyo mwachezganga nayo, navinyakhe). |

### Interview Guide for PMTCT Focal persons (Tumbuka)

| **Mundondomeko wakendesyero kavidumbirano pakuchezga: Balalabalala abo bakwendeska ntchito yavya PMTCT pachipatala.**  Kafukufuku wa NEMAPP |
| --- |
| Kuchezga **#: Boma:**  **Chipatala/malo**  Zuba ilo mukanjirira mukafukufuku (Zuba) (Mwezi)    Nambala yakafukufuku wa NEMAPP  **Vyaka vya uyo akuzgola:**  **Ngwakutengwa:**  1= ali panthengwa  2= mfumu wake alukufwa  3= balulekana nthengwa  4= bandatengwepo kawiro  5= vinyakhe (phalani)________________________________  **Masambiro**  1= Palije/wandayeko kusukulu  2= Wakalekera makalasi ghakusika ku primary  3= Wakalekera makalasi ghakuchanya ku primary  4= Wakalekera makalasi ghakusika ku secondary  5= Wakarekera makalasi ghakuchanya ku secondary  6= Walupanga masambiro ghapachanya/masambiro gha ukachenjede  7= Masatifiketi ghanyakhe namadipuloma (zunulani)____________________  8= Vinyakhe (zunulani)_______________________________________________  **Mutundu**  1= Muyao  2= Mulomwe  3= Muchewa  4= Mutumbuka  5= Mngoni  6= Musena  7= mutundu unyakhe (zunulani)______________________  **Chipembezo**  1= Mukhilisitu  2= Musilamu  3= Chabapapi/chaukhaliro  4= Palinje panji vinyakhe (zunulani)______________________  **Mwanalume/Mwanakazi:**  **Wakufumba/wakwendeska vidumbirano:**    **ZUBA/MWEZI/CHILUMIKA**    **HRS**  **HRS**  **Zuba: Nyengo yakwambira: Nyengo yakumalira:** |
| **Vyakwenera kulondezga uyo wafumbenge mafumbo:**   - Londezgani mundondomeko wa chipepala chakulongola kuzomerezga kutolapo lwande mukafukufuku uyu, kweniso fumbani kuti bamuzomerezgani kujambula vidumbirano ivi. Mama uyo muchezgenge nayo uyo, mupasani nyengo yakukwanira kuti aghanaghane makola ghene. Munthu uyo muchezgenge nayo, abe wakufwasa/kusutuka pavidumbirano ivi ndipo muchezge kwa mphindi 60 (mungamanya kunozgaso ulendo unyakhe kuti muzakachezge nayo usange panyengo yasono nivyamachitiko yaye). - Ni udindo winu kuwonesyeska kuti kuchezga kwinu muvidumbirano ivi nikwamuchindindi/chisisi (kusazgirapo navyose ivyo vyajambulikanga pala mwadumbirananga). - Iyi nindondomeko yakendesyero kavidumbirano ivyo makani ghakhe pala muchezga mulongosolerana mwakufikapo khanira (nkhufubana waka mafumbo nopasana mazgoro yayi): fumbisiskani mwakufikapo pa mazgoro agho bakumuzgorani kuti bamuphalirani mwakupulikikwa makola ghene kweniso sandanisandani maghanoghano ghabo mwakufika khanira. Kuchezga kwinu kube nge mudumbirana munthu namunyakhe kuluska nakuti vibe nge mufubana waka mafumbo nopasana mazgoro nge nimumayeso. - Kufumira apa kuluta munthazi, kuchezga kwinu muvidumbirano ivi kujambulike, nachizomerezgo kufuma kwa abo mutichezgenge nabo. - Lembani ivyo mwaviwona kuti nivyakukhumbikwa/vyachandulo panyengo yeneyiyo kuumaliro kwa pepala ili, pazuba leneliro la kuchezga kwinu. |
| 1. Kasi maghanoghano ghinu ghakuti ghuli pachipwelerero cha PMTCT icho chuperekeka pachipatala mu boma ili?   *[Fumbani]*: Ivyo vikwenda makola nivichi kweniso ivyo vikwenera kusitha nivichi?   1. Kasi mungalongosolapo ivyo bakusangana navyo bamama abo bakupokera ghovyiri wachipwelerero cha PMTCT pachipatala mu boma ili?   *[Fumbani]:* Kasi banakazi/bamama abo bakutorapo chipwererero cha PMTCT, balumuphaliranipo mazgu ghalighose panji kukhwafyika kulikose pakuyana na ntchito ya PMTCT?   1. Mumaghanoghano ghinu, kasi nivichi ivyo vikuchitika panji vingachitikanga nachakulata chakuti badada/banalume nabo batolengepo lwande pachiipwelerero cha PMTCT? 2. Kasi ghulipo ghovyiri ghulighose ugho mukughuwona kuti ghukusobekera? Panji pali ghovyiri wakukhumbikwa kwa bamama namabanja ghabo kuti uperekekenge? 3. Kasi mukughughona ghuli mundondomeko na umo ulili/ukwendera pala bakwambiska balwali pa munkhwala wa HIV, chomenechomene kwa bamama abo bakupokera ghovyiri wa PMTCT?   *[Fumbisiskani]:* Mumaghanoghano ghinu, kasi nivichi ivyo vikwenera kusitha pa mundondomeko ugho bakulondezga pakumwambiska munthu munkhwala?   1. Mazgu gha ‘Option B+’ mukubamanya? Para mukubamanya, mukugomezga kuti ghakung’anamura vichi?   *[Fumbisiskani]:* Kasi pulogilamu iyi yikusithangapo vichi pamakani ghakukhwafyana na ghovyiri wamunkhwala wa HIV, kupimiska ndopa, kokheska bana kweniso pamakani ghakulimbikiska badada/banalume kutolapo lwande pa PMTCT?   1. Kasi mukuyanayana kuti kuba paghovyiri wa munkhwala wa HIV kungiza nachandulo chilichose pamakani ghakokheska bana mu boma lino?   *[Fumbisiskani]: pangaba chandulo ghuli pamakani ghakokheska bana pakuyana nakuba paghovyiri wakupokera munkhwala wa HIV, ndipo ninjani uyo ukukhwafyika chomene?*   1. Kasi mughanaghana kuti kuba paghovyiri wamunkhwala wa ART kungaba nachandulo kuti bamama babe namaghanoghano ghakutolaso nthumbo zinyakhe mu boma lino?   *[Fumbisiskani]: kasi pangaba chandulo ghuli pamakani ghakutolaso nthumbo inyakhe pakuyana nakuba paghovyiri wakupokera munkhwala wa ART, ndipo ninjani uyo ukukhwafyika chomene?*   1. Mumaghanoghano ghinu, kasi mukughanaghana kuti nivifukwa ghuli ivyo vikupangiska bamama kuleka kwizaso kuzakatola munkhwala panji kwizaso pa zuba labo lakubikika muvipatala vya mu boma ili?   *[Fumbisiskani]: mukuwona kwinu, nivifukwa ghuli ivyo bamama bakulekera kumwa munkhwala? Kasi mundondomeko wa chipwererero cha PMTCT, wendesyekenge ghuli kuti bamama balimbiskike kwizaso kuchipatala kuzakapoka ghovyiri wamunkhwala nachipwererero cha HIV?*   1. Mumaghanoghano ghinu, kasi mukughona nge nighovyiri ghuli wapachanya ugho ungapasikanga kwa bamama panji abo banamukhoza/nthumbo mu boma ili?   *[Fumbisiskani]: tiphalirani umoza mwa maghovyiri pakuyana nachiphwererero cha PMTCT agho ghangakhumbikwa kuperekekanga mu boma ili?*   1. Mu boma ili, kasi makani ghakukhwafyana na HIV ghakubikika kuba ghamuchindindi/chisisi? Kasi makani gha HIV ghakuyoghoyekanga mwakufikapo?   *[Fumbisiskani]: kasi chomenechomene mukuphwerererapo makani ghuli ghakukhwafyana na HIV ndpo mukumalananga nagho ghuli makani nge ni agha? Kasi mu mwezi panji vyaka vyajumpha ivi, mukughanaghana kuti pali kusitha kulikose pakuyana nakugegana pakukhwafyana nabanthu abo bali nakachibungu ka HIV? Pala kulipo kusitha kulikose, chonde phalani. Pala palije kusitha kulikose, longosolani kuti kasi nichifukwa ghuli palije kusitha.*   1. Mukuwona kwinu, kasi vilipo vyakujanda vilivyose ivyo vingapangiska bamama abo bali nakachibungu ka HIV kuba poghovyiri wa chiphwererero cha PMTCT?   *[Fumbisiskani]:* longosolani vyakujanda pala viripo? Kasi viri nachandulo ghuli mu boma ili? Kasi nivichi ivyo vingalutiska panthazi mulimo/ntchito yakupimiska ndopa pakukhwafyana nakachibungu ka HIV naghovyiri wamunkhwala?   1. Kasi nizifundo ghuli izo ziluvyirapo bamama abo bali nakachibungu ka HIV mu boma ili kupokera ghovyiri wamunkhwala nachiphwererero pakukhwafyana na HIV?   *[Fumbisiskani]: longosolani zifundo izi pala zilipo? Kasi zilupindulirapo ghuli bamama mu boma ili?* |
| Pambere tindajale vidumbirano vithu, kasi muli nandemanga/vyakuyowoyapo virivyose vyakukhwafyana namakani agho tadumbirananga pakuchezga kwithu? [longosolani] |
| **Vyakwenera kuchita uyo ofumba mafumbo:**   - Bawongani abo bamuzgoraninge mafumbo/mwachezganga nabo chifukwa chakutola nyengo yabo nakutolapo lwande pakafukufuku uyu. Bakumbuskani bamama aba kuti bangazomera, muzamukwizaso navidumbirano nge ni ivi pala pajumpha mwezi 6. - Pala mwamala vidumbirano vinu (zuba leneriro) lembani musika umu vyose ivyo mwavighonanga kuti vikayovyire kusazgirapo pa uthenga ugho watolereka pavidumbirano ivi (vingaba nge malo, umo vidumbirano vyayenderanga, vyakutimbanizga ivyo vyangubapo, umo kuchezga/mafumbo ghenderanga, umo angubira/apulikiranga pavidumbirano ivi, kuleka kufwasa/kujiwezga pakuyowoya pavidumbirano, umo alili uyo mwachezganga nayo, navinyakhe). |
